# Domain-wall magnetoelectric coupling in multiferroic hexagonal YbFeO_3_ films

**DOI:** 10.1038/s41598-023-28365-x

**Published:** 2023-01-31

**Authors:** Xin Li, Yu Yun, Arashdeep Singh Thind, Yuewei Yin, Qiang Li, Wenbin Wang, Alpha T. N’Diaye, Corbyn Mellinger, Xuanyuan Jiang, Rohan Mishra, Xiaoshan Xu

**Affiliations:** 1 Department of Physics and Astronomy, University of Nebraska, Lincoln, NE 68588 USA; 2 Institute of Materials Science and Engineering, Washington University in St. Louis, St. Louis MO, USA; 3 Institute for Nanoelectronic Devices and Quantum Computing, Fudan University, Shanghai, 200433 China; 4 Advanced Light Source, Lawrence Berkeley National Laboratory, Berkeley, CA 94720 USA; 5 Department of Mechanical Engineering and Materials Science, Washington University in St. Louis, St. Louis, MO USA; 6 Nebraska Center for Materials and Nanoscience, University of Nebraska, Lincoln, NE 68588 USA

**Keywords:** Magnetic properties and materials, Ferroelectrics and multiferroics

## Abstract

Electrical modulation of magnetic states in single-phase multiferroic materials, using domain-wall magnetoelectric (ME) coupling, can be enhanced substantially by controlling the population density of the ferroelectric (FE) domain walls during polarization switching. In this work, we investigate the domain-wall ME coupling in multiferroic h-YbFeO_3_ thin films, in which the FE domain walls induce clamped antiferromagnetic (AFM) domain walls with reduced magnetization magnitude. Simulation according to the phenomenological theory indicates that the domain-wall ME effect is dramatically enhanced when the separation between the FE domain walls shrinks below the characteristic width of the clamped AFM domain walls during the ferroelectric switching. Experimentally, we show that while the magnetization magnitude remains same for both the positive and the negative saturation polarization states, there is evidence of magnetization reduction at the coercive voltages. These results suggest that the domain-wall ME effect is viable for electrical control of magnetization.

## Introduction

Ferroelectricity and magnetism, originating from different symmetry conditions^1–3^, have been considered as independent phenomena mostly. However, over the past decades, ME effects, with various coupling mechanisms between electrical and magnetic properties, were revealed in both composite materials and single-phase materials via the interfaces and the intrinsic interplay of charge, spin, and crystal structures respectively^4–8^. Among various ME effects, the electrical control of magnetization is especially appealing for information storage and processing applications due to the scalability and high energy efficiency^9^.

For a single-phase multiferroic materials, the effect of bulk-state polarization reversal on the magnetization, i.e., bulk ME effect, is expected to be weak, since the inversion of crystal structure, as required by polarization reversal, does not change the preferred spin direction necessarily within individual domain. Alternatively, ME effects can be triggered by FE domain walls if they are coupled to the magnetic domain walls. Therefore, by controlling the polarization switching, the domain-wall ME effect may be effective for electric-field modulation of magnetization.

**Figure 1:**
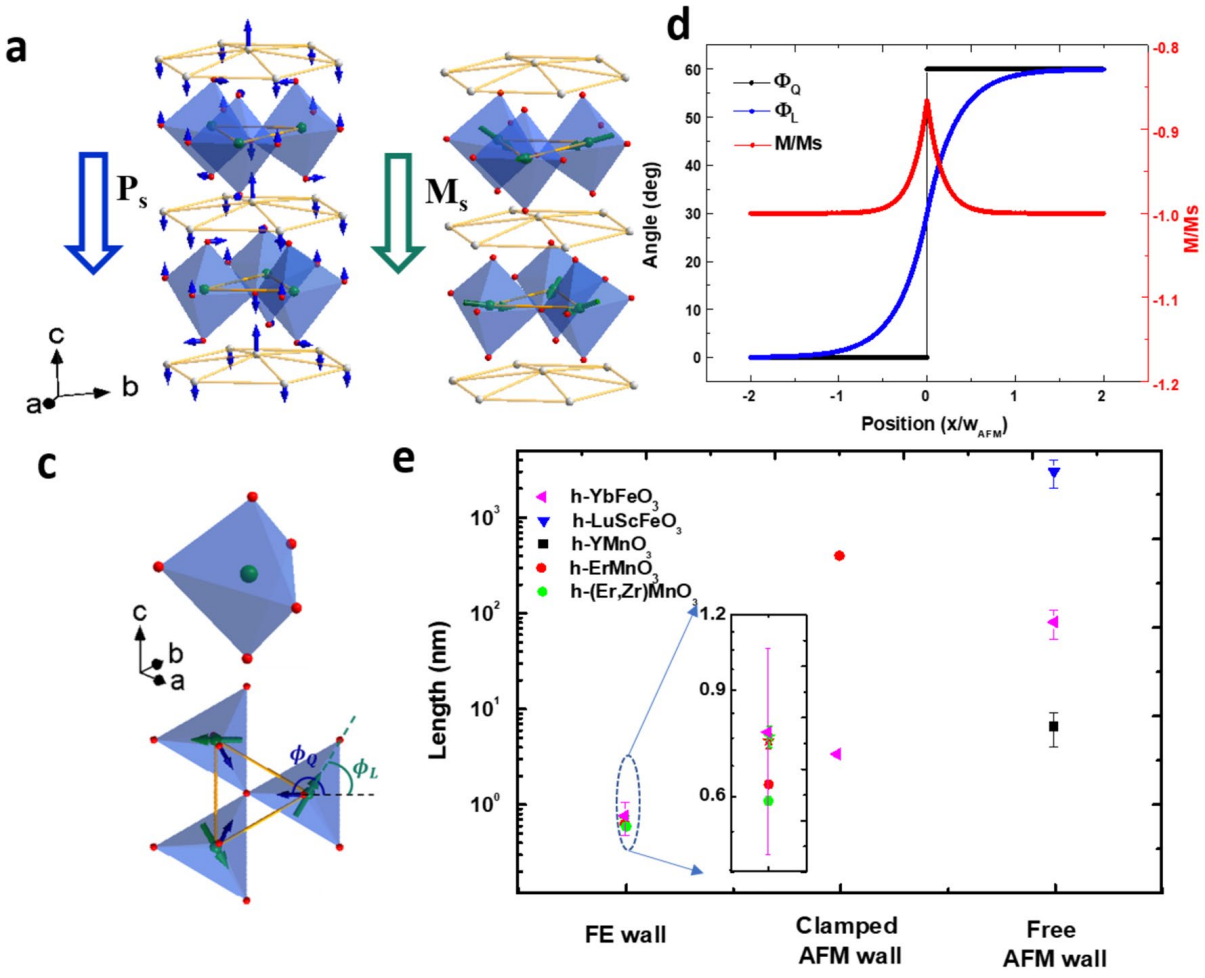
(**a**) Atomic structure and K_3_ structural distortion of h-*R*FeO_3_. Green, white, and red spheres represent Fe, rare-earth, and O atoms, respectively. The arrows indicate the displacement of the O and the Yb atoms of the K_3_ mode. (**b**) Schematic illustration of weak ferromagnetism (or canted antiferromagnetism) of h-*R*FeO_3_, the green arrows through the Fe atoms represent the spin directions. (**c**) FeO_5_ bipyramids and the definition of order parameters ${\phi }_{Q}$ and ${\phi }_{L}$. (**d**) Schematic profiles of ${\phi }_{Q}$, ${\phi }_{L}$ and normalized magnetization at the clamped AFM wall in h-YbFeO_3_, based on numerical simulation (see text). (**e**) Comparison of characteristic widths of FE wall, clamped and free AFM walls in multiferroic hexagonal ferrites and manganites, the inset is the close-up view of the data of the FE walls. Data for h-(Lu,Sc)FeO_3_, YMnO_3_, h-ErMnO_3_ and h-(Er,Zr)MnO_3_ comes from Ref. 11, 23, 28, 31, respectively. The width of the clamped AFM wall of h-YbFeO_3_ is from the simulation in (**d**). The width of free AFM wall of h-YbFeO_3_ is from the fitting of frequency shift of MFM image (see Fig. S13).

Here we focus on multiferroic hexagonal ferrites (h-*R*FeO_3_, *R* = Y, Sc, Ho-Lu), whose complex interplay between ferroelectricity, magnetism and structural distortion^10,11^, especially the spin–lattice coupling, may enable substantial domain-wall ME effects. The crystal structure of h-*R*FeO_3_ consists of layers of FeO_5_ trigonal bipyramids separated by layers of *R* ions, as shown in Fig. 1a. Similar to the isomorphic hexagonal manganites (h-*R*MnO_3_)^12–16^, h-*R*FeO_3_ exhibits improper ferroelectricity with non-centrosymmetric *P*6_3_cm structure, which are induced by the K_3_ structural distortion below ~ 1000 K. As illustrated in Fig. 1a, the K_3_ structural distortion can be viewed as the collective tilt of the FeO_5_, which causes buckling of the *R* layer and induces the spontaneous polarization ($P$) along the *c* axis^12,17,18^. Below the Neel temperature (~ 150 K)^19,20^, h-*R*FeO_3_ becomes antiferromagnetic with the Fe spins mostly lying in the basal plane with an 120° order. The tilt of FeO_5_ in the K_3_ distortion leads to the canting of Fe spins due to the spin–lattice coupling manifested in single-ion magnetic anisotropy (SIA) and the Dzyaloshinskii-Moriya (DM) interaction^21^, generating the spontaneous magnetization along the *c* axis, namely, canted antiferromagnetism^19,22^, as shown in Fig. 1b. The direction of the FeO_5_ tilt can be represented by an in-plane angle ${\phi }_{Q}$, and the in-plane direction of Fe spins can be described by an in-plane angle ${\phi }_{L}$ within the same coordinate (see Fig. 1c). Moreover, when magnetic ions, such as Yb^3+^, occupy the *R* sites, their magnetic moments are aligned by the exchange field from the neighboring Fe spins, leading to the enhancement of magnetization^19,20^.

Since both the ferroelectric order and the weak ferromagnetic order are induced by the K_3_ structural distortion, h-*R*FeO_3_ is promising for the ME effect. Although the bulk ME effect is negligible due to the mismatched length scale of the ferroelectric domains and that of the weak ferromagnetic domains^23^, substantial domain-wall ME effect is expected. Across the FE domain wall, the change of ${\phi }_{Q}$ leads to the change of ${\phi }_{L}$, as required by the SIA, generating a so-called clamped antiferromagnetic (AFM) domain wall whose magnetization is different from the bulk value. Therefore, the domain-wall ME effect offers a way to modulate magnetization by controlling the population density of the FE domain walls. This mechanism applies to both h-*R*MnO_3_ and h-*R*FeO_3_. While for h-*R*MnO_3_, the clamped AFM wall has non-zero magnetization in contrast to the zero bulk value^24–28^, for h-*R*FeO_3_, the magnitude of the magnetization is reduced from the bulk value in the clamped AFM walls (see Fig. 1d). Despite being appealing for electric control of magnetization^23,28,29^, the domain-wall ME effect in h-*R*FeO_3_ has not been systematically investigated either theoretically or experimentally, especially about the role of the large mismatch between the width of the FE domain wall and that of the clamp AFM domain wall (see Fig. 1e).

In this work, we combine the phenomenological model and the experimental studies to reveal the key factors that determine the domain-wall ME effect in multiferroic h-YbFeO_3_ thin films. It was found that the domain-wall ME effect could be greatly enhanced by achieving small FE wall separation (high population density) during polarization switching. Meanwhile, larger width of the FE walls reduces the domain-wall ME effect, but to a smaller extent. Experimentally, broadened FE walls and related distribution of structural order parameters (Q, ${\phi }_{Q}$) were identified under multidomain state in h-YbFeO_3_ thin films of small grains. Through in-situ polarization switching, the reduction of magnetization at coercive voltage and the subsequent recovery at saturated polarization states demonstrate the electrical modulation of magnetization by the domain-wall ME effect in h-YbFeO_3_ films.

## Results

### Epitaxial growth and crystal structure of h-YbFeO_3_ films

**Figure 2:**
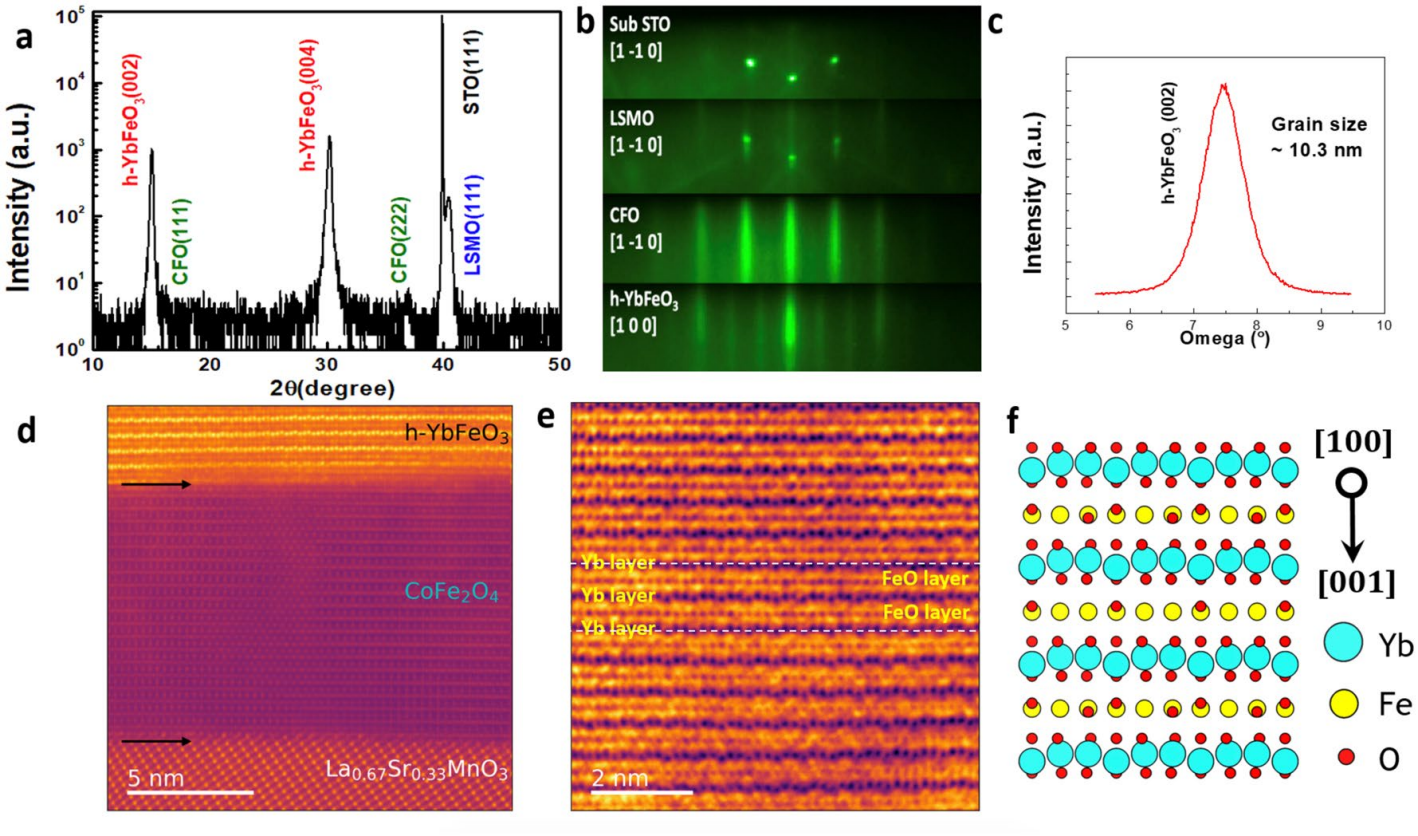
(**a**) *θ*-2*θ* XRD scan of h-YbFeO_3_/CFO/LSMO/STO(111) films. (**b**) RHEED patterns and in-plane epitaxy relationship of individual layer, with electron beam along the h-YbFeO_3_[100] direction. (**c**) Rocking curve of the h-YbFeO_3_ (002) peak. (**d**) Cross-section HADDF-STEM of h-YbFeO_3_/CFO/LSMO/STO(111)films, along the h-YbFeO_3_ [100] direction. (**e**) ABF images of single ferroelectric domain area of h-YbFeO_3_. (**f**) Schematic alignment of individual atoms within h-YbFeO_3_ along the [100] direction.

High-quality h-YbFeO_3_/CFO/LSMO heterostructures were designed and fabricated on STO(111) substrates by pulsed laser deposition (PLD), in which the LSMO (La_2/3_Sr_1/3_MnO_3_) layer is used as the bottom electrode, and the CFO (CoFe_2_O_4_) layer serves as a buffer layer to reduce the lattice mismatch between the LSMO layer and the h-YbFeO_3_ layer, stabilizing the *P*6_3_cm structure of h-YbFeO_3_. To illustrate the structure of the h-YbFeO_3_ films, x-ray diffraction (XRD), reflection high energy electron diffraction (RHEED), and scanning transmission electron microscopy (STEM) measurements were carried out. Figure 2a shows the representative *θ*-2*θ* XRD scan of h-YbFeO_3_/CFO/LSMO/STO heterostructure without obvious impurity phases. As shown in Fig. 2b, sharp and clear streaks of the h-YbFeO_3_ layer indicate smooth surfaces and the in-plane epitaxial relationship between individual layers. Along the STO [–211]/(111) orientation, the intense diffraction streaks separated by the weak streaks in the RHEED pattern of the h-YbFeO_3_ layer are indicators of the polar *P*6_3_cm structure. Figure 2c shows the rocking curve of the h-YbFeO_3_ (002) peak, indicating grain size of 10.3 nm according to Scherrer formula. Figure 2d shows a wide field-of-view high-angle annular dark-field (HAADF) image of the h-YbFeO_3_/CFO/LSMO/STO heterostructure viewed along the h-YbFeO_3_[001] direction, where the two arrows indicate the atomically sharp h-YbFeO_3_/CFO and CFO/LSMO interfaces, respectively. In the annular bright-field (ABF) image of a single ferroelectric domain region in the h-YbFeO_3_ layer (Fig. 2e), the buckling of the Yb layers indicates that the polarization is pointing up, where the neighboring Yb layer and FeO layer within one unit cell are labeled. The atomic model of h-YbFeO_3_ viewed along the [100] direction is given in Fig. 2f.

### Switching paths for the ME coupling and decoupling in h-YbFeO_3_

**Figure 3:**
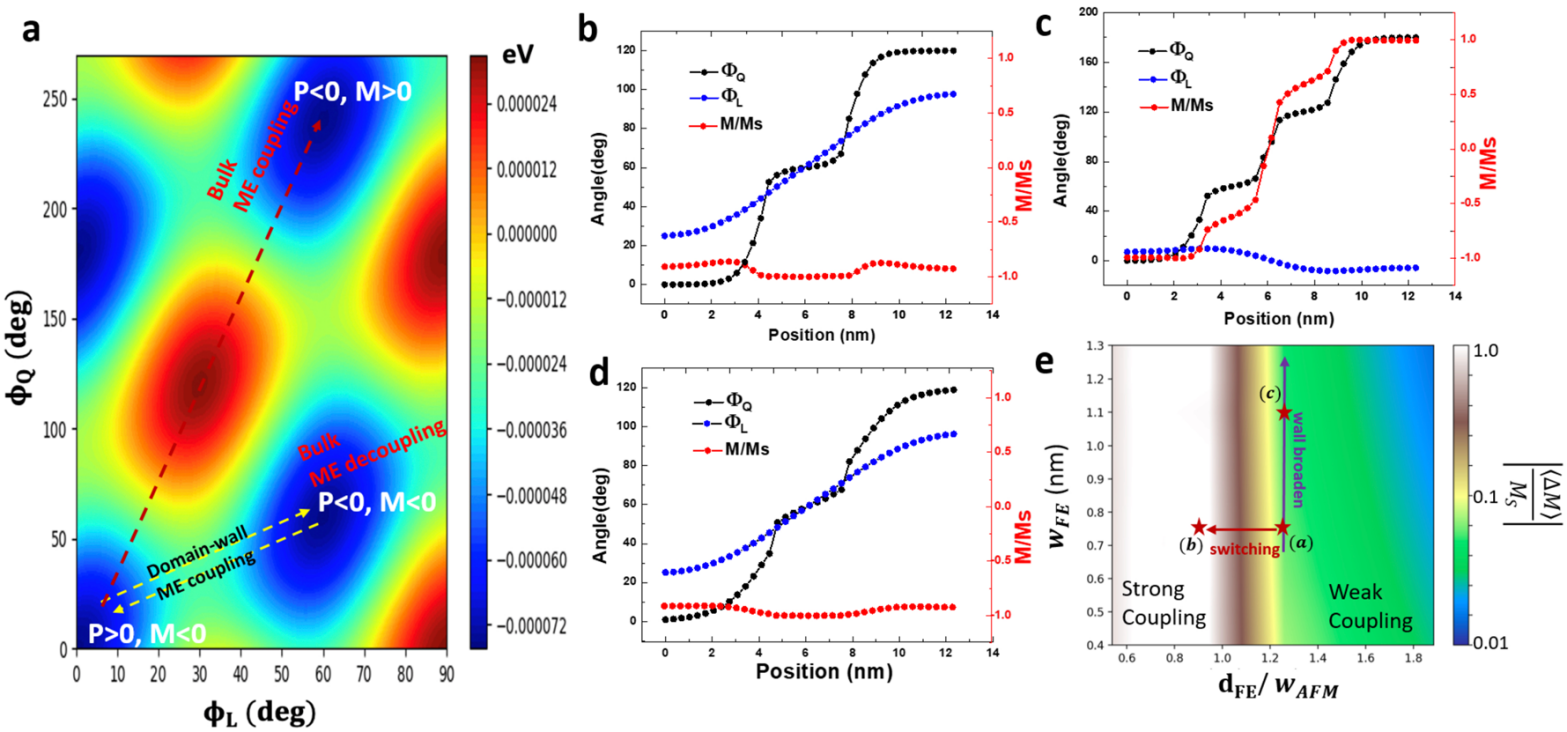
(**a**) Landscape of magnetic free energy of h-YbFeO_3_ with ${\phi }_{Q}$ and ${\phi }_{L}$ as independent parameter, with arrows correspond to potential switching paths for ME coupling and decoupling. (**b**–**d**) The domain-wall ME coupling inside a 12.5 nm grain with multiple FE walls and corresponded profiles of ${\phi }_{Q}$, ${\phi }_{L}$ and M/Ms. The FE wall width is 0.75 nm in (**b**,**c**) and 1.1 nm in (**d**). (**e**) Phase diagram of averaged reduction of magnetization due to domain-wall ME coupling, with respect to w_FE_ and d_FE_/w_AFM_, the red and purple arrows represent polarization switching and FE wall broadening in h-YbFeO_3_ films.

To illustrate the available switching paths for both bulk and domain-wall ME coupling effects in h-YbFeO_3_, the energy landscape is calculated based on the Landau theory with ${\phi }_{Q}$ and ${\phi }_{L}$ as independent parameters, as shown in Fig. 3a. In h-*R*FeO_3_, canted spins of the Fe ions induce spontaneous magnetization along the *c*-axis, which depends on the relative orientation between ${\phi }_{Q}$ and ${\phi }_{L}$ as $M=-{M}_{s}\mathrm{cos}({\phi }_{Q}-{\phi }_{L})$, where ${M}_{s}$ is the magnitude of saturated magnetization. Within a single FE domain, ${\phi }_{Q}$ takes the value of $n\frac{\pi }{3}$ (*n*: integer)^11^; SIA requires ${\phi }_{Q}-{\phi }_{L}=n\pi$, corresponding to the A_2_ magnetic structure in h-*R*FeO_3_. As shown in Fig. 3a, along the polarization switching path indicated by the red arrow, the magnetization is also switched, corresponding to the bulk ME coupling. However, this path is unlikely due to the high energy barrier. Along the energy-favorable polarization switching path (yellow arrows), the magnetization state remain the same for the initial and the final states, suggesting bulk ME decoupling (see details in supplementary S1). However, polarization switching is often accompanied by the formation and motion of the FE domain walls. Their effect on magnetization can be substantial when the population density of the FE walls is high.

### Domain-wall ME coupling in h-YbFeO_3_ films based on phenomenological model

To analyze the important factors that determine the domain-wall ME coupling in h-YbFeO_3_, we start with the modulation of magnetization within a single clamped AFM wall. Across an FE domain wall, ${\phi }_{Q}$ changes by $\pm \frac{\pi }{3}$, so does ${\phi }_{L}$ due to the SIA, generating the clamped AFM wall. In Fig. 1d, based on the structural-distortion-related magnetic free energy, the ${\phi }_{L}$ profile is calculated across a sharp FE domain wall (${\phi }_{Q}$ profile is a step function, see supplementary Sect. S2). The width of the clamped AFM domain wall can be described as ${w}_{AFM}=\pi \sqrt{\frac{S}{|A|}} ,$ where with *S* and *A* are exchange stiffness and anisotropy constant.^11^ Using the parameters in Table S1^11,25^, we found ${w}_{AFM}\approx$ 3.3 nm. Far away from the FE domain wall, the matching of ${\phi }_{Q}$ and ${\phi }_{L} ({\phi }_{Q}-{\phi }_{L}=0$) leads to undisturbed magnetization ($|M|={M}_{s}$). However, within the clamped AFM domain wall, $\left|{\phi }_{Q}-{\phi }_{L}\right|\ne n\pi$ causes the change of magnetization ($\Delta M=M-Ms$), which is essential for the electrical modulation of magnetization of the domain-wall ME coupling.

Considering the larger population of the FE domain walls during the polarization switching, the modulation of average magnetization $$\frac{\langle \Delta M\rangle }{{M}_{S}}$$ may be dramatically enhanced when the FE domain size or the separation between the FE domain walls ($${d}_{FE})$$ is smaller than $${w}_{AFM}.$$ Here we use a one-dimensional model in which the parallel neutral-type FE domain walls are evenly distributed within a single crystallite with fixed size of 12.5 nm with the free boundary condition. As shown in Fig. 3b, the small separation between the FE domain walls requires $${\phi }_{L}$$ to change more rapidly than that in Fig. 1d. Consequently, the change of $${\phi }_{L}$$ can barely follow that of $${\phi }_{Q}$$, causing an enhanced $$\left|\frac{\langle \Delta M\rangle }{{M}_{S}}\right|$$. More importantly, as shown in Fig. 3c, when the $${d}_{FE}$$ is further reduced, $${\phi }_{L}$$ decouples from $${\phi }_{Q}$$ and remains approximately constant, regardless of the change of $${\phi }_{Q}$$. As a result, $$\frac{M}{{M}_{S}}$$ varies dramatically between − 1 and 1. On average, $$\left|\frac{\langle \Delta M\rangle }{{M}_{S}}\right|$$ approaches 1 (or $$\frac{\langle M\rangle }{{M}_{S}}$$ approaches 0), corresponding to total quenching of average magnetization due to the proliferation of FE walls. A similar trend for quenching magnetization can also be identified when the FE wall number is fixed but the crystallite size decreases (see Figs. S3–S5). Therefore, when the separation between FE wall is smaller than $${w}_{AFM}$$, the large $$\left|\frac{\langle \Delta M\rangle }{{M}_{S}}\right|$$ corresponds to a significant domain-wall ME effect, and the proliferation of FE walls during polarization switching may facilitate the spatial distribution of FE walls to reach the critical condition ($${d}_{FE}<{w}_{AFM}$$) for enhanced domain-wall ME effect.

In contrast to the separation of the FE wall ($${d}_{FE})$$, the width of the FE wall $${(w}_{FE}$$) influences the domain-wall ME effect to a much smaller extent. Typically, the width of FE domain walls is less than 1 nm. However, geometrical constrictions could increase the volume fraction of the FE domain walls or $${w}_{FE}$$^16,30^. Since the width mismatch between the FE domain wall and the clamped AFM wall is the key, the broadening of the FE wall is expected to reduce the mismatch between $${\phi }_{L}$$ and $${\phi }_{Q}$$ and reduce the domain-wall ME effect. As shown in Fig. 3d, in a crystallite with two FE domain walls, when the FE wall gets wider than that in Fig. 3b, the more gradual change of $${\phi }_{Q}$$ makes it easier for $${\phi }_{L}$$ to follow, and the deviation of $$|M|$$ from $${M}_{s}$$ then becomes smaller than that in Fig. 3b, as indicated by the profile of M/Ms. Therefore, the broadening of $${w}_{FE}$$ would slightly reduce the domain-wall ME effects.

### Phase diagram of magnetization reduction for domain-wall ME coupling

To illustrate the competing effect of ${d}_{FE}$ and ${w}_{FE}$ on the domain-wall ME coupling, the phase diagram of $\left|\frac{\langle \Delta M\rangle }{{M}_{S}}\right|$ as a function of ${d}_{FE}$ and ${w}_{FE}$ is displayed in Fig. 3e, in which the dashed and solid arrows represent increased FE domain wall population density and wall broadening respectively. For sparse distribution of FE domain walls or ${d}_{FE}/{w}_{AFM}\gg 1$, the change of $\left|\frac{\langle \Delta M\rangle }{{M}_{S}}\right|$ is small, corresponding to the weak-coupling region. When ${d}_{FE}$ approaches ${w}_{AFM}$ (along the dashed arrow), the domain-wall ME effect transits from the weak-coupling region to the strong-coupling region where, Broader FE wall does weaken the ME coupling, but this effect is much smaller than and it mainly influences the domain-wall ME coupling in the weak-coupling region. Therefore, to achieve experimental observable electrical modulation of $\frac{\langle \Delta M\rangle }{{M}_{s}}$, it is essential to have small ${d}_{FE}$ or high population density of the FE domain walls. Typically, ferroelectric switching starts from the nucleation of reversed polarization which generates FE domain walls, followed by the propagation and coalescence of these walls, and the high density of domain walls is more likely to exist in ferroelectrics films comprised of small crystallites where the boundaries or related defects hinder the free motion of FE domain walls, favoring the nucleation limited switching (NLS) mode during polarization reversal^32–34^.

### The distribution of ferroelectric domain wall and structural order parameters

**Figure 4:**
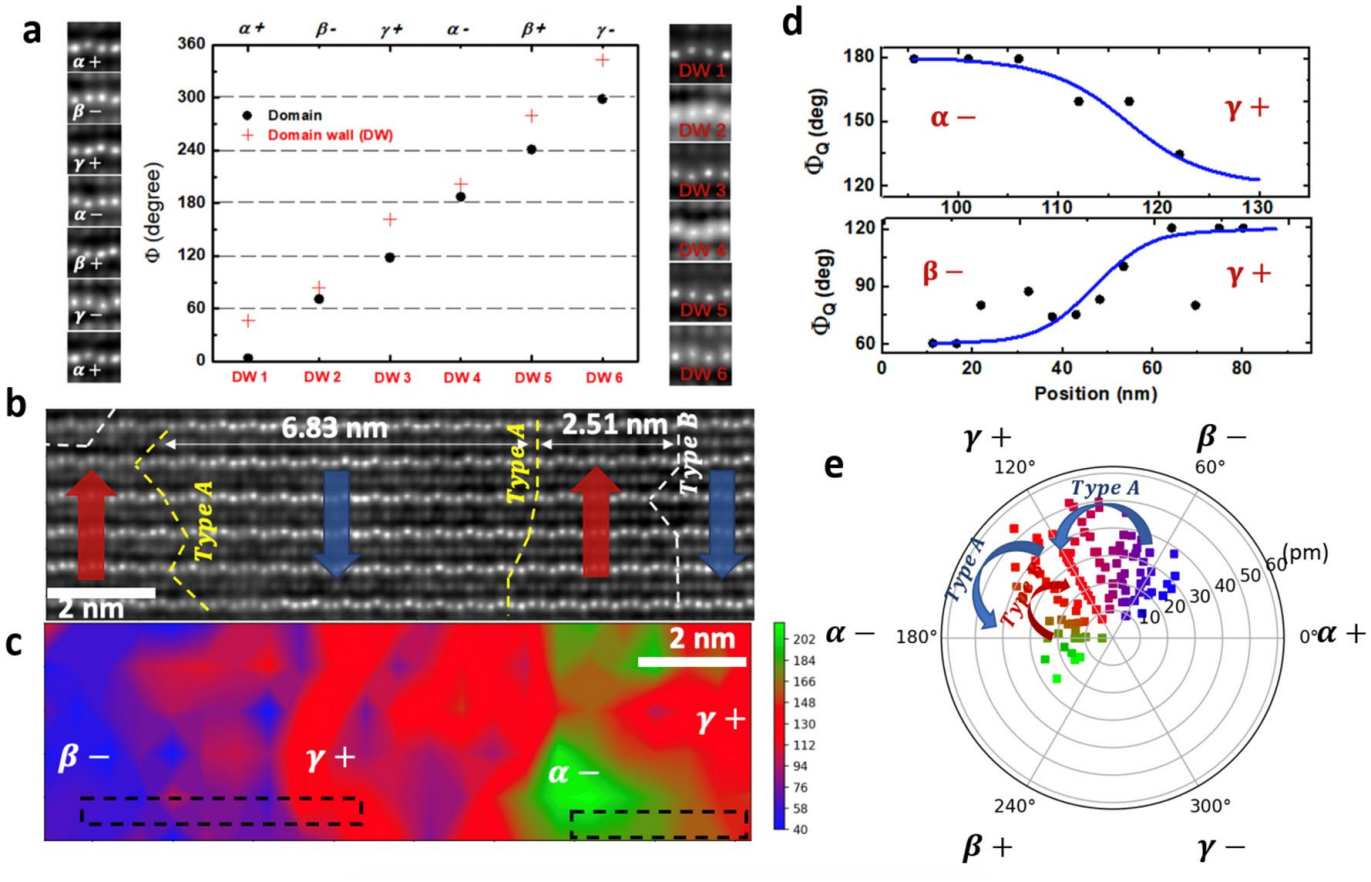
The distribution of FE domain walls and structural order parameters in h-YbFeO_3_ film with small crystallites. (**a**) Characteristic four-atom patterns and ${\phi }_{Q}$ distribution for individual FE domains and domain walls based on the HADDF images. (**b**) Cross-section HADDF-STEM of multidomain area in h-YbFeO_3_ films along the [100] zone axis. (**c**) The spatial distribution of ${\phi }_{Q}$ corresponding to (**b**). (**d**) Line profiles of $\alpha -|\gamma +$ and $\beta -|\gamma +$ FE domain walls in dashed boxes of (**c**). (**e**) The pole figure of the structural order parameters (Q, ${\phi }_{Q}$) distribution for the FE multidomain area, based on the HADDF image of (**b**).

To visualize the distribution of the FE domain walls inside small grains, the displacements of Yb ions were analyzed quantitatively using HADDF-STEM images with picometer precision (see details in supplementary Sect. S4). The schematic patterns of Yb ions for the FE domains (${\phi }_{Q} =\frac{n\pi }{3}$ ) and domain walls (${\phi }_{Q} =\frac{2n+1}{6}\pi$), obtained from the HADDF images, is shown in Fig. 4a. The corrugation pattern of the FE domain walls can be considered as the superposition of atom positions from the neighboring FE domains, therefore, there also exist six characteristic patterns for FE domain walls, providing the basis to quantify ${\phi }_{Q}$ for the intermediate states around the FE walls. Figure 4b displays an HADDF image for a multidomain region in the h-YbFeO_3_ layer. Along the [100] zone axis, six layers of trimerized Yb lattice can be identified and there exists five FE domains with polarization directions indicated by the arrows. The positions of the FE domain walls are highlighted by the dot lines, where the most of them belong to neutral wall along the <001> direction. The extracted ${\mathrm{d}}_{\mathrm{FE}}$ is 6.83 nm between two the type-A walls, and 2.51 nm between the type-A and the type-B wall. Therefore, d_FE_ partially satisfies the critical condition (${d}_{FE}/{w}_{AFM}$<1) for strong domain-wall ME coupling in Fig. 3e; the average d_FE_ is expected to further decrease at coercive voltage where *P* equals zero. The spatial distribution of ${\phi }_{Q}$ is displayed in Fig. 4c, indicating the broadened ${w}_{FE}$. Related line profiles of ${\phi }_{Q}$, inferred from the atom positions of Yb, are shown in Fig. 4d, and the characteristic width are determined as 12 $\pm 0.4$ Å based on the ${\phi }_{Q}$ profile from the Landau theory^31^, which is different from the ideal model of atomic-sharp FE walls considered in previous work^11,36^, but is consistent with the broaden FE walls in thin film and superlattice^30,31^ (see details in supplementary Sect. S4). Moreover, the relation between the magnitude $(Q)$ and the direction (${\phi }_{Q}$) of the K_3_ structural distortion are plotted by a pole figure in Fig. 4e, confirming a large volume fraction of intermediate phase with ${\phi }_{Q} \ne \frac{n\pi }{3}$ which belongs to the FE domain walls. Therefore, within the individual grains of epitaxial h-YbFeO_3_ films, there exists stripe-like domains with broadened FE domain walls, and ${d}_{FE}$ could partially satisfy the critical condition for achieving substantial domain-wall ME coupling.

### Experimental evidence of domain-wall ME coupling during polarization switching

**Figure 5:**
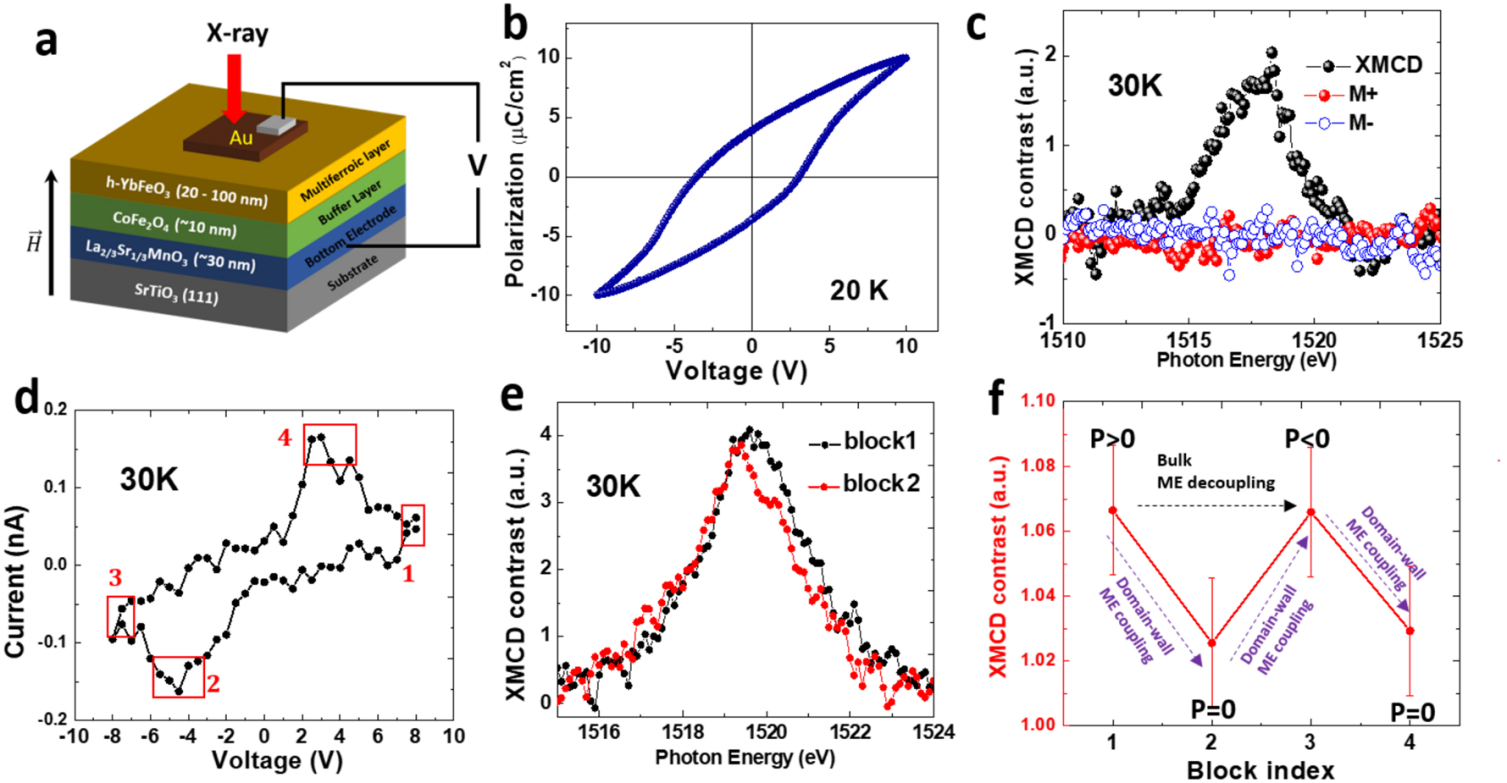
(**a**) Schematic diagram of the measurement setup. (**b**) Ex-situ ferroelectric hysteresis loop of h-YbFeO_3_ films. (**c**) The remnant XMCD spectrum and the contrast of x-ray absorption before and after the polarization reversal for both remanent magnetization states (M + and M −) of the Yb M_5_ edge. (**d**) In-situ measurement of switching current with voltage in h-YbFeO_3_ film during XMCD measurement at 30 K. The blocks 1–4 correspond to the range of voltage for averaging the XMCD signals. (**e**) Remanent XMCD spectra of Yb M_5_ edge when the film is under positive saturated polarization (block 1) and multidomain state near negative coercive voltage (block 2). (**f**) Integrated XMCD contrasts at the Yb M_5_ edge and polarization states during different stages of ferroelectric switching.

To examine the enhancement of domain-wall ME coupling effect mentioned above, the magnetization of Yb ions were measured using x-ray absorption spectroscopy (XAS) and x-ray magnetic circular dichroism (XMCD) during in-situ polarization switching, as illustrated by the schematic diagram in Fig. 5a. The ex-situ polarization-voltage (*P*–*V*) hysteresis loop, measured at 20 K, demonstrates that coercive voltages are around $\pm 4V$ (see Fig. 5b). The magnetization reversal was demonstrated using remanent XMCD by measuring the contrast of absorption spectra near the Yb M_5_ edge with a circularly polarized x-ray in zero magnetic field after applying $\pm$ 18 kOe field, as shown in Fig. 5c. The switching path for bulk ME decoupling can be inferred from the minimal contrast of x-ray absorption for remanent magnetization states (M + and M −) before and after the polarization reversal (see details in supplementary Sect. S5).

The domain-wall ME effect is measured by comparing the XMCD spectra of Yb ions at different stage of polarization switching. As shown in Fig. 5d, the in-situ polarization switching is verified by the voltage-current hysteresis loop, where peaks of switching current occur at the coercive voltage (≈ $\pm$ 4 V), consistent with the ex-situ measurements. The blocks 1 to 4 correspond to the voltage ranges for averaging the XMCD signals. The blocks 1 and 3 correspond to the positive and the negative saturated polarization states respectively; block 2 and 4 are near coercive voltage, where the film is expected to be in multidomain state (*P* = 0) with highest density of FE domain walls. The detailed XMCD spectra for individual voltages between $\pm 9V$ are given in supplementary, with each condition being measured multiple times to reduce the uncertainty. As shown in Fig. 5e, within the photon energy between 1519 to 1524 eV, the XMCD contrast of Yb M_5_ edge undergoes observable reduction near the negative coercive voltage (block 2), comparing with the contrast of positive polarization state (block 1). The detailed comparison for the four blocks is given in Fig. S13. Moreover, as shown in Fig. 5f, the XMCD contrast recovers for the negative polarization state (block 3) and reduces again near the positive coercive voltage (block 4), indicating the robustness of the domain-wall ME effect during the polarization switching.

## Discussion

Based on the polarization and magnetization states of each block in Fig. 5f, the black arrow indicates that the observable switching path in h-YbFeO_3_ film is indeed the one for bulk ME decoupling, as indicated by low-barrier switching path in Fig. 2a. Moreover, the reproducible magnetization reduction at both negative (block 2) and positive coercive voltage (block 4), where the polarization is close to zero, corresponds to the electrical field driven magnetization modulation via the domain-wall ME effect discussed above. These results provide the first experimental evidence for electrical modulation of magnetization in h-RFeO_3_ films by domain-wall ME coupling on the switching path favoring bulk-state ME decoupling.

The domain-wall ME effect may be tuned by thickness and temperature in the h-RFeO_3_ films. In particular, in the heterostructure studied here, reduction of the h-RFeO_3_ film thickness may promote the multidomain state before reaching the ultrathin limit^32^. The corresponding higher population density of the FE walls may further enhance the domain-wall ME effect. Since the ME effect relies on both the ferroelectric and the magnetic orders, the temperature range under discussion is limited by T_N_ ~ 120 K for h-YbFeO_3_. In this range, the ferroelectric properties are not expected to change substantially according to the Curie temperature Tc (~ 1000 K)^37^. The thermal-driven change for the domain-wall ME effect is expected to come mainly from the variation of magnetic properties such as magnetic anisotropy and exchange stiffness. More in-situ studies of the clamped AFM walls are necessary to reveal the temperature effects.

In summary, based on the phenomenological theory, we have shown that the domain-wall ME effect can be strong enough to turn the spontaneous magnetization on and off in multiferroic h-YbFeO_3_ thin films when the distribution of FE domain wall reaches the critical condition (${d}_{FE}/{w}_{AFM}$<1). Experimentally, by measuring the remanent magnetization during the in-situ polarization switching, we have observed the indication of magnetization reduction at the coercive voltage where the density of FE domain wall is the maximum. These results suggest that even if the switching path of bulk ME decoupling is energy favorable, the domain-wall ME effect could still be a viable route to realize the control of macroscopic magnetization in multiferroic h-RFeO_3_ films by polarization switching.

## Methods

### Thin film growth

The h-YbFeO_3_ thin films (20–100 nm thick) were grown on CoFe_2_O_4_ /La_2/3_Sr_1/3_MnO_3_/SrTiO_3_ (111) and yttrium stabilized zirconia (YSZ) (111) substrates by pulsed laser deposition (PLD) system with a KrF excimer laser (248 nm and 2 Hz repetition rate), at the growth temperature from 650 to 850 °C and oxygen pressure of 10 mTorr. Before thin-film deposition, substrates were pre-annealed at 700 °C for 1 h. The La_2/3_Sr_1/3_MnO_3_ (LSMO) layer (~ 30 nm) was grown at a substrate temperature of 700 °C and oxygen pressure of 80 mTorr on the SrTiO_3_ (STO) substrate. The CoFe_2_O_4_ (CFO) layer (~ 10 nm) was grown at the temperature of 600 °C and the oxygen pressure of 10 mTorr. The film growth was monitored using in-situ reflection high-energy electron diffraction (RHEED). The Au (3–5 nm) top electrodes were evaporated by an AJA sputtering system with 300–400 μm diameter.

### Structural characterization

The structural phase of the epitaxial films was determined using X-ray diffraction (XRD) (Rigaku SmartLab). Scanning transmission electron microscopy (STEM) imaging was carried out using the aberration-corrected Nion UltraSTEMTM 200 microscope (operating at 200 kV) at Oak Ridge National Laboratory. An electron transparent thin foil for STEM characterization was prepared using a Hitachi NB5000 focused ion and electron beam system. To protect against the ion beam damage, a 1-μm-thick carbon layer was deposited on top of the h-YbFeO_3_ film surface. A 20 kV beam with a current of 0.7 nA was used to cut the lift-out. Rough and fine milling were performed at 10 kV and 5 kV with beam currents of 0.07 nA and 0.01 nA respectively. The resulting foil was mounted on a Cu grid, which was baked at 160 ℃ under vacuum prior to the STEM experiments to remove surface contamination.

### Magnetic domain measurement

A commercial AFM/MFM (Atto AFM/MFM Ixs; Attocube Systems) was used to map the topography and magnetic images at 20 K. During the measurement, the MFM was performed in constant height mode (single pass) with PPP-MFMR tip from NANOSENSORS. The lift height is 100 nm. The resolution of the image in supplementary is 60 nm, and the scan speed is 1um/s.

### XMCD measurements

The X-ray absorption spectroscopy (XAS) (including X-ray magnetic circular dichroism or XMCD) was studied at the beamline 6.3.1 in the Advanced Light Source at Lawrence Berkeley National Laboratory.

### Ferroelectric measurements

The FE polarization was switched by the DC current using a Keithley 236 source meter (the measurements of high-resolution I-V curves) and a Keithley 2450 source meter (in-situ polarization switching during XMCD measurements). The polarization versus electric filed (P–V) loops were measured using a Precision RT66C Ferroelectric Tester.

## Supplementary Information

Supplementary Information.

## Data Availability

The datasets used and/or analyzed during the current study available from the corresponding author on reasonable request.
