# Supplementary material for: Domain-wall magnetoelectric coupling in multiferroic hexagonal YbFeO3 films
Source: Sci Rep. 2023 Jan 31;13:1755. doi: 10.1038/s41598-023-28365-x (PMC9889801; doi:10.1038/s41598-023-28365-x)
Supplement: Supplementary file 1 — Supplementary Information. [file 41598_2023_28365_MOESM1_ESM.docx]

Supplementary material:

Domain-wall magnetoelectric coupling in multiferroic hexagonal YbFeO_3_ films

*Xin Li,^1^*^‡^ *Yu Yun,^1^*^‡^** Arashdeep Singh Thind,^2^ Yuewei Yin,^1^ Qiang Li,^3^ Wenbin Wang,^3^ Alpha T. N’Diaye,^4^ Corbyn Mellinger,^1^ Xuanyuan Jiang,^1^ Rohan Mishra,^2,5^ Xiaoshan Xu^1,6^**

^1^Department of Physics and Astronomy, University of Nebraska, Lincoln, Nebraska 68588, USA

^2^Institute of Materials Science & Engineering, Washington University in St. Louis, St. Louis MO, USA

^3^Institute for Nanoelectronic Devices and Quantum Computing, Fudan University, Shanghai 200433, China

^4^Advanced Light Source, Lawrence Berkeley National Laboratory, Berkeley, California 94720, USA

^5^Department of Mechanical Engineering & Materials Science, Washington University in St. Louis, St. Louis MO, USA

^6^Nebraska Center for Materials and Nanoscience, University of Nebraska, Lincoln, Nebraska 68588, USA

‡These authors contributed equally to this work.

*Corresponding author: xiaoshan.xu@unl.edu (X.X.) yunyu1110@gmail.com (Y.Y.)

Table of Contents

[Section S1: Bulk-state ME decoupling in h-RFeO_3_ from Landau theory. 2](#_Toc103086850)

[Section S2: Domain-wall ME coupling of clamped AFM wall in h-RFeO_3_ 4](#_Toc103086851)

[Section S3: Crystal structure characterization 9](#_Toc103086852)

[Section S4: Quantitative analysis of HADDF-STEM images 10](#_Toc103086853)

[Section S5: XECD and XMCD measurements 12](#_Toc103086854)

[Section S6: Low temperature MFM measurements of h-YbFeO_3_/YSZ films. 13](#_Toc103086855)

# Section S1: Bulk-state ME decoupling in h-RFeO_3_ from Landau theory

To understand bulk-state ME decoupling mechanisms in h-YbFeO_3_, a phenomenological model based on Landau theory of free energy is considered. The structural distortion and the spin configuration can be described using $\phi_{Q}$ and $\phi_{L}$, which correspond to the title angle of FeO_5_ (or displacement angle of the apex oxygen) and the in-plane Fe spin orientation in the same FeO_5_, respectively. The free energy in the Landau theory related to the K_3_ structural distortion (magnitude Q, phase angle $\phi_{Q}$) and polarization (P) can be written as

$f_{1}=\frac{a}{2}Q^{2}+\frac{b}{4}Q^{4}-gQ^{3}P cos(3\phi_{Q})+\frac{g'}{2}Q^{2}P^{2}+\frac{a_{p}}{2}P^{2}+\frac{s}{2}\left[ \left( \nabla Q \right)^{2}+Q^{2}\left( \nabla\phi_{Q} \right)^{2} \right]$ (1)

The energy contribution of K_3_ structural distortion comes from the first two terms, while the third and the fourth terms represent the non-linear coupling between K_3_ structural distortion and the polar $\Gamma_{2}^{-}$ structural distortion whose amplitude is proportional to the polarization, the last stiffness terms account for the energy of spatially varied K_3_ structural distortion. The equilibrium states, corresponding to the energy minima of f_1_, locate at $\phi_{Q}= n\frac{\pi}{3}$ (n = 0, 2, … 5). Minimizing f_1_ with respect to P, leads to the dependence of P on $\phi_{Q}$:

$P=\frac{gQ^{3}cos(3\phi_{Q})}{g'Q^{2}+a_{P}}$ (2)

As illustrated in Figure. S1(a), for constant Q, each polarization state corresponds to three $\phi_{Q}$ values (three structural distortion configurations).

Without considering the existence of domain walls, the magnetic free energy, assuming a fixed alignment between the angles $\phi_{Q}$ (and $\phi_{L}$) on two FeO_5_ layers at z = 0 and z = c/2 in the unit cell, can be written as^11^

$f_{2}=\left( A+C_{+} \right)\cos\left[ 2\left( \phi_{L}-\phi_{Q} \right) \right]$ (3)

This equation combines the single-ion magnetic anisotropy ^21^ and the interlayer-interaction ^20^; the Zeeman energy is omitted assuming the absence of an external magnetic field. The magnetization depends on the canting angle which is determined by the alignment between $\phi_{L}$ and $\phi_{Q}$:

$M=-\cos\left( \phi_{Q}-\phi_{L} \right)M_{s}$ (4)

where $M_{s}$is the magnitude of the saturated magnetization.

Based on structural and magnetic free energy, bulk-state ME decoupling can be proven using $\phi_{Q}$ and $\phi_{L}$ as independent parameters, and the effects of domain walls are not considered. According to Eq. (2) and (3), each polarization state corresponds to three structural distortion configurations (three $\phi_{Q}$ values, see Figure. S1(a) and Eq. 3), and that each $\phi_{Q}$ corresponds to two equal-energy spin directions described by $\phi_{L}=\phi_{Q}$ and $\phi_{L}=\phi_{Q}+\pi$ corresponding to opposite magnetization directions according to Eq.(5). So, there are 12 minima in the free-energy ($f_{1}+f_{2}$) landscape in the ($\phi_{Q}, \phi_{L}$) space, as shown in Figure. S1b (Q = 0.33 Å). After polarization reversal, there are six possible final spin-structure configurations, corresponding to six paths in the ($\phi_{Q}, \phi_{L}$) space. As depicted in Figure. S1(c), for the paths that change $\phi_{Q}-\phi_{L}$, magnetization is also changed after polarization reversal, meaning magnetization and polarization are coupled; otherwise, the magnetization remains the same after the polarization reversal, meaning that magnetization and polarization are decoupled. The question is which path is the most energy favorable.

According to Figure.S1 (b), the paths $\left\{ \phi_{Q}=\phi_{L}=0 \right\}$ ⟶ $\left\{ \phi_{L}=\phi_{Q}=\pm\frac{\pi}{3} \right\}$ has the lowest energy barrier, which holds when the amplitude Q increases (see Figure. S2). Since the $\phi_{Q}-\phi_{L}$ does not change in this lowest-barrier path, magnetization remains the same [see Eq. (4)] after the polarization reversal, i.e., the magnetization and polarization are decoupled. This is corresponding to the vanishing bulk-state ME coupling in h-RFeO_3_.


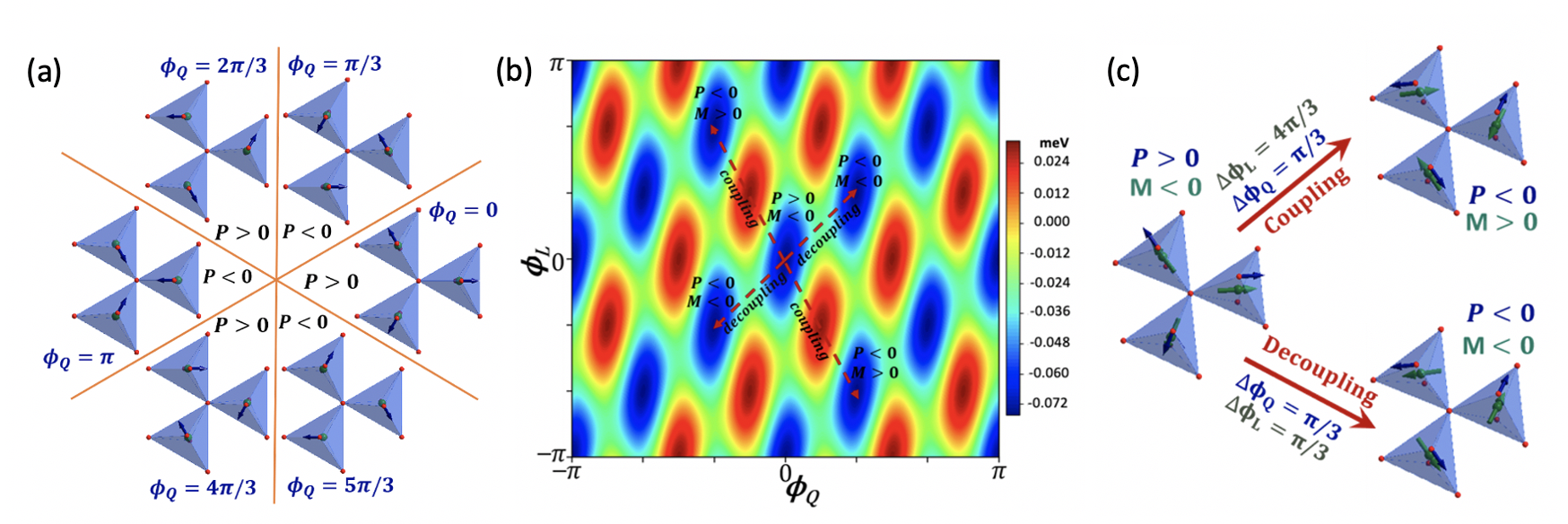


**Figure. S1.** Bulk ME decoupling in h-RFeO_3_. (a) Six $\phi_{Q}$ states with positive and negative polarizations. (b) Free-energy landscape in the $(\phi_{Q},\phi_{L})$ space, where Q is fixed at 0.33 Å. (c) Schematic illustration of switching (spin-structure) paths with and without magnetization reversal in the single-domain mechanism in response to polarization reversal.


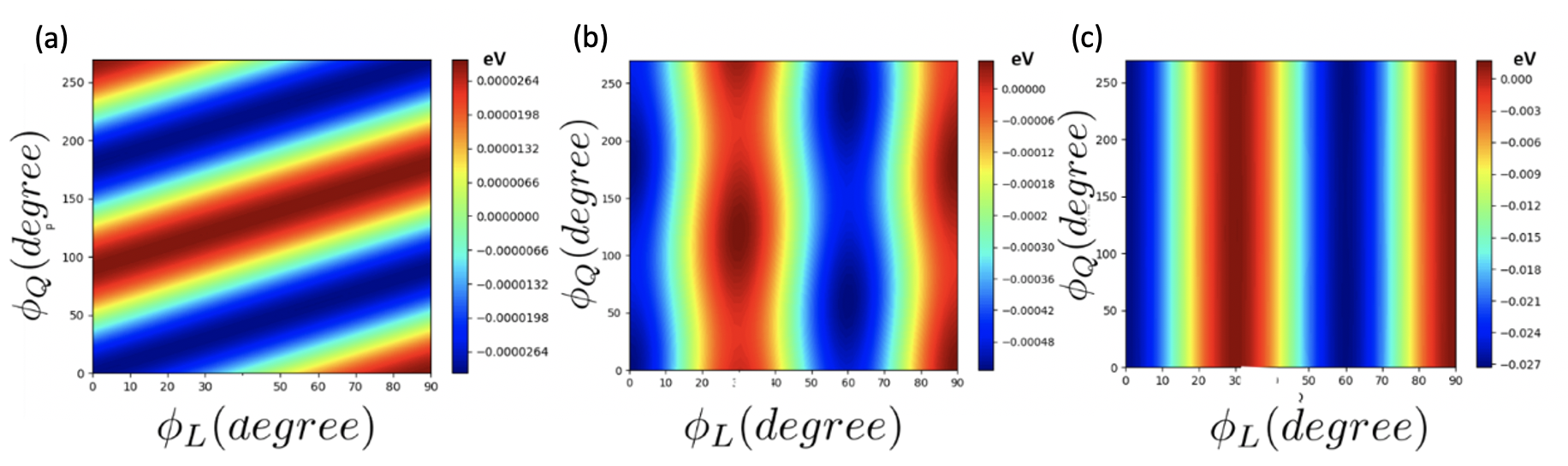


**Figure. S2.** Energy landscape with various Q. Energy landscape with Q is fixed at (a) 0.1 Å, (b) 0.5 Å, and (c) 1.0 Å, respectively.

# Section S2: Domain-wall ME coupling of clamped AFM wall in h-RFeO_3_

Section S2.1 Equation of order parameter $\phi_{L}$

The spatial variation of order parameter $\phi_{L}$ at the clamped AFM domain wall is determined by the exchange stiffness term and in-plane anisotropy in magnetic free energy, which can be expressed as:

$f_{\mathrm{mag}}\left( \phi_{L} \right)=\left( A+C_{+} \right)\cos\left( 2\left( \phi_{L}-\phi_{Q}(x) \right) \right)+2S\left( \frac{{\partial\phi}_{L}}{\partial x} \right)^{2}$ (5)

in which $A$describes from in-plane anisotropy, $C_{+}$describes inter-layer exchange interaction, S describes exchange stiffness.

Total energy of magnetic domain wall is:

$\sigma=\int_{-\infty}^{+\infty} \left[ \left( A+C_{+} \right)cos\left( 2\left( \phi_{L}-\phi_{Q}(x) \right) \right)+2S\left( \frac{{\partial\phi}_{L}}{\partial x} \right)^{2} \right]dx$ (6)

Then, for equilibrium state:

$\delta\sigma=\int_{-\infty}^{+\infty} \left[ \left( A+C_{+} \right)\frac{\partial cos\left( 2\left( \phi_{L}-\phi_{Q}(x) \right) \right)}{\partial\phi_{L}}-4S\frac{{\partial^{2}\phi}_{L}}{\partial x^{2}} \right]\delta\phi_{L}dx + |4S\frac{{\partial\phi}_{L}}{\partial x}{\delta\phi}_{L}|=0$ (7)

Therefore,

$\left( A+C_{+} \right)\frac{\partial cos\left( 2\left( \phi_{L}-\phi_{Q}(x) \right) \right)}{\partial\phi_{L}}-4S\frac{{\partial^{2}\phi}_{L}}{\partial x^{2}}=0$ (8)

Next step is doing integration:

$\int_{-\infty}^{+\infty} \frac{{\partial\phi}_{L}}{\partial x}\left[ \left( A+C_{+} \right)\frac{\partial cos\left( 2\left( \phi_{L}-\phi_{Q}(x) \right) \right)}{\partial\phi_{L}}-4S \frac{\partial}{\partial x}\frac{{\partial\phi}_{L}}{\partial x} \right]dx=0$ (9)

Then,

$\left( A+C_{+} \right)cos\left( 2\left( \phi_{L}-\phi_{Q}(x) \right) \right)-2S {(\frac{{\partial\phi}_{L}}{\partial x})}^{2}=const$ (10)

For boundary condition at $x=\infty$

$\phi_{L}=\phi_{Q}(x)$， $\frac{{\partial\phi}_{L}}{\partial x}=0$， so $const=A+C_{+}$

So,

$\frac{{\partial\phi}_{L}}{\partial x}=\pm\left( \frac{\left( A+C_{+} \right)}{2S}cos\left( 2\left( \phi_{L}-\phi_{Q}\left( x \right) \right) \right)-\frac{\left( A+C_{+} \right)}{2S} \right)^{\frac{1}{2}}$ (11)

$\partial x=\pm\left( \frac{\left( A+C_{+} \right)}{2S}cos\left( 2\left( \phi_{L}-\phi_{Q}\left( x \right) \right) \right) \right)^{-\frac{1}{2}}{\partial\phi}_{L}$ (12)

Based on this equation, we can get the numerical solution of $\phi_{L}$(x) under stripe-like FE domain states with the profile $\phi_{Q}\left( x \right)$.

The magnetic structures of clamped AFM wall were calculated from the magnetic free energy of equation (13), with parameters as below:

| S(meV$\cdotÅ^{2}$) | A(meV) | C_+_(meV) |
| --- | --- | --- |
| 22 | -0.02 | 0 |

**TABLE S1**: Parameters of magnetic free energy Eq. (6) per 1 f.u.

Section S2.2 Effect of FE wall number and crystallite size on clamped AFM wall

In this part, the width of FE domain wall was fixed at 0.75 nm, we use the equation derived in Section 2.1 to solve the profiles of $\phi_{L}$(x) numerically under stripe FE domain states inside one grain, as shown in Figure. S3. Based on the magnetization profile (Figure. S4), it is obvious when the grain size is small enough and number of FE wall is large enough across the critical condition ($d_{FE}/w_{AFM}\approx$1), the M/Ms can change from -1 to 1 inside the clamped AFM wall, corresponding to stronger magnetization reduction. This enhancement originates from that the virtually $\phi_{L}$(x) regardless of the $\phi_{Q}$(x) profile.

|  | 5 nm | 10nm | 20 nm |
| --- | --- | --- | --- |
| 1 | 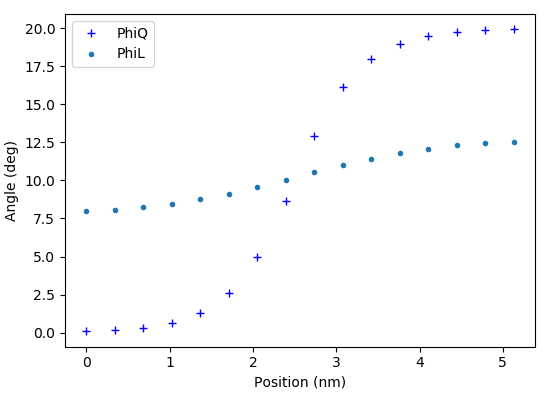 | 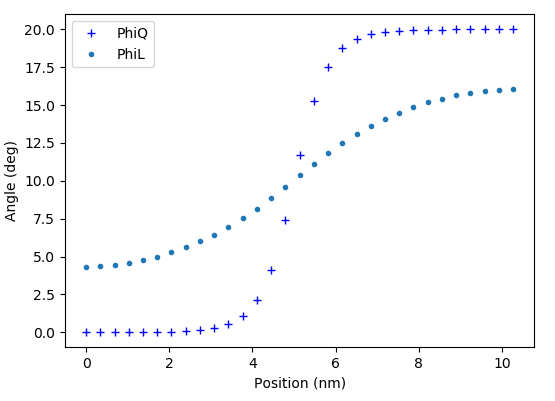 | 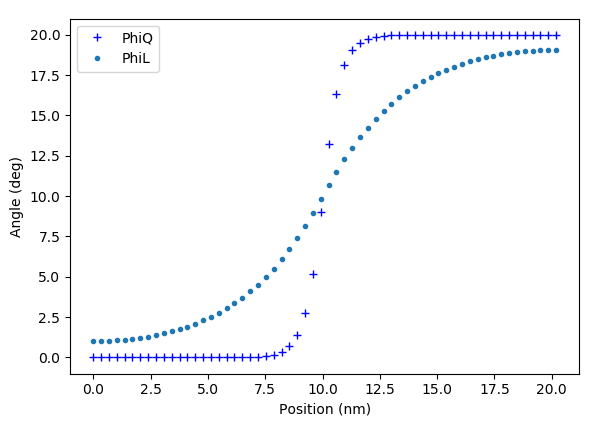 |
| 2 | 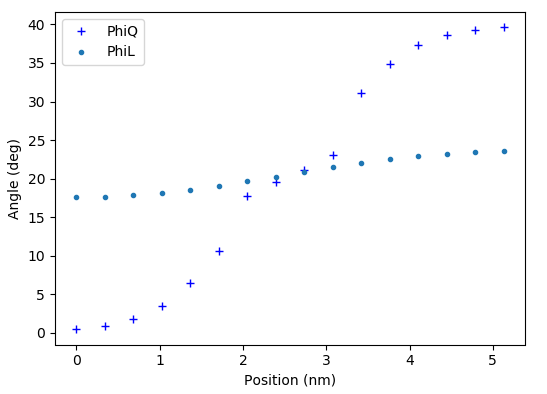 | 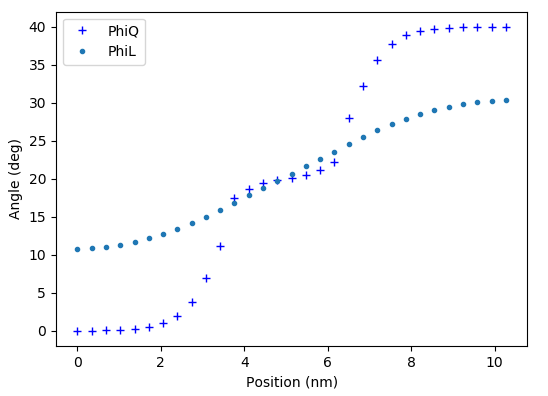 | 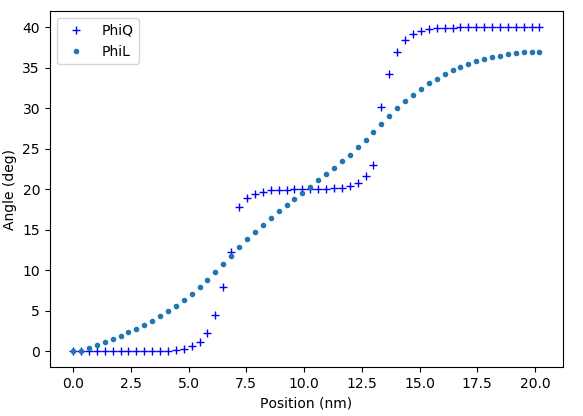 |
| 3 | 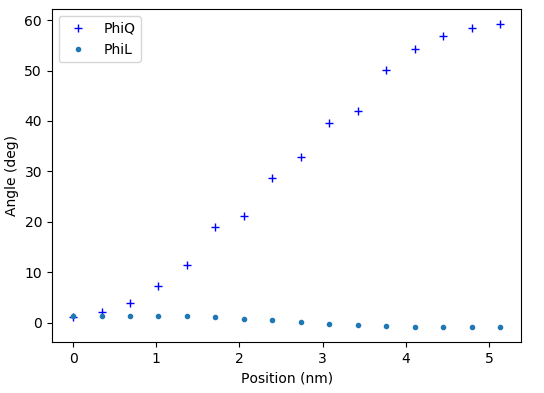 | 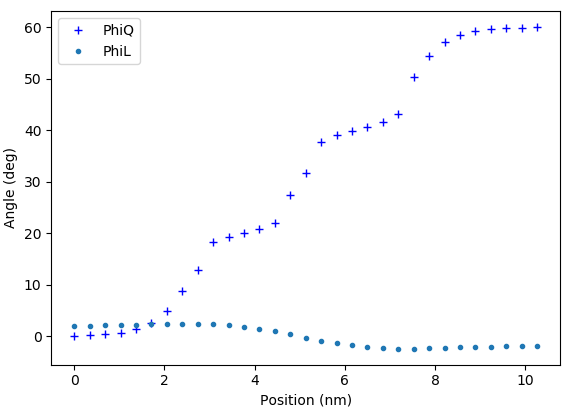 | 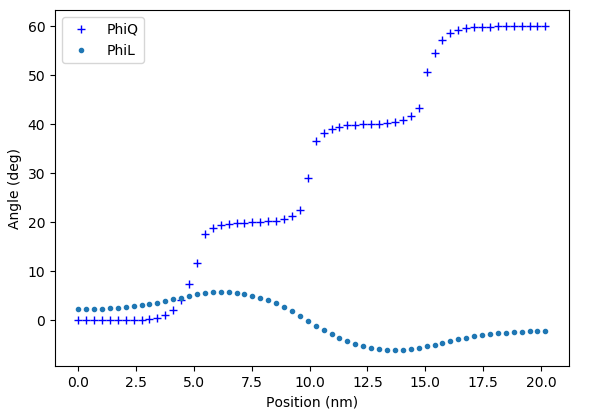 |
| 4 |  | 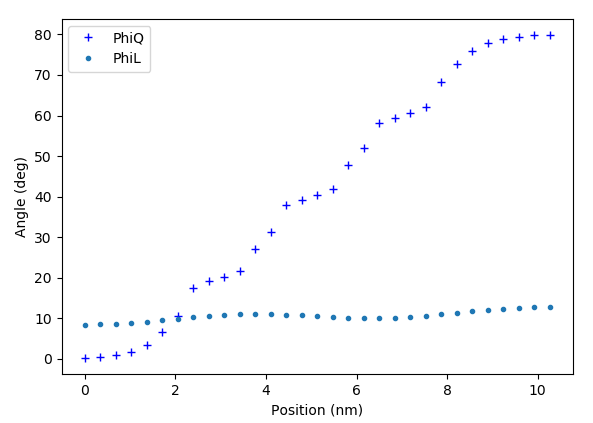 | 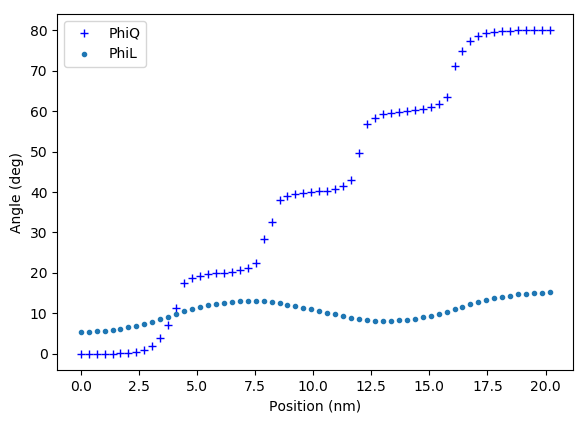 |
| 5 |  |  | 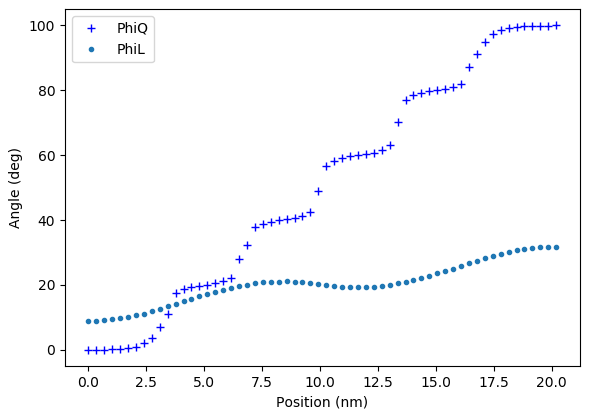 |

**Figure. S3** $\phi_{L}$ and $\phi_{Q}$ profiles under different grain size and FE wall number, the width of FE wall was fixed at 0.75 nm.

|  | 5 nm | 10nm | 20 nm |
| --- | --- | --- | --- |
| 1 | 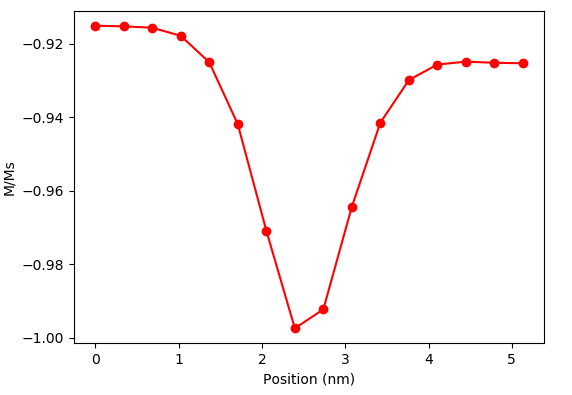 | 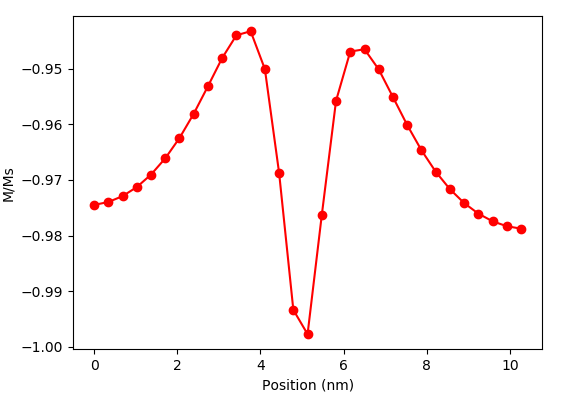 | 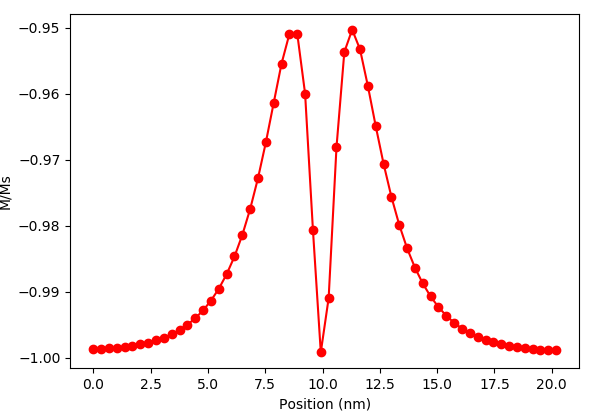 |
| 2 | 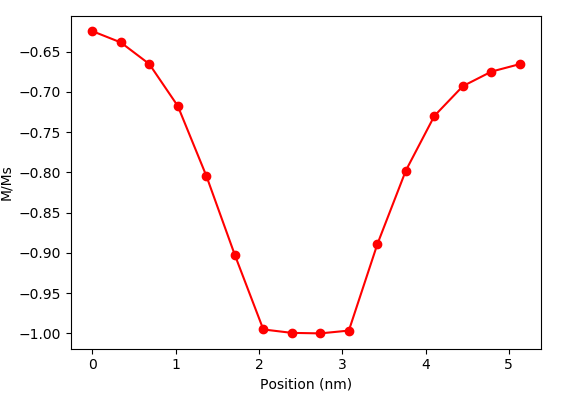 | 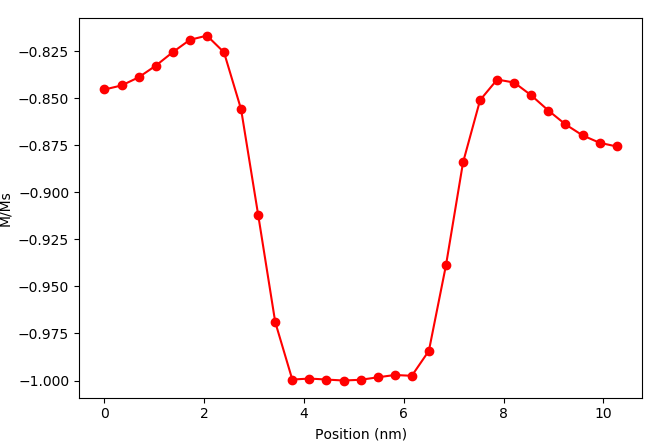 | 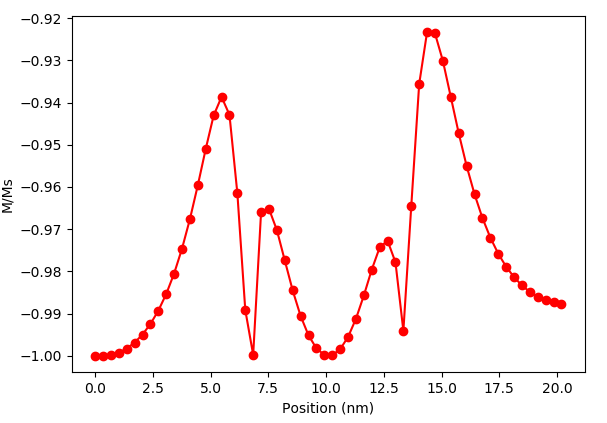 |
| 3 | 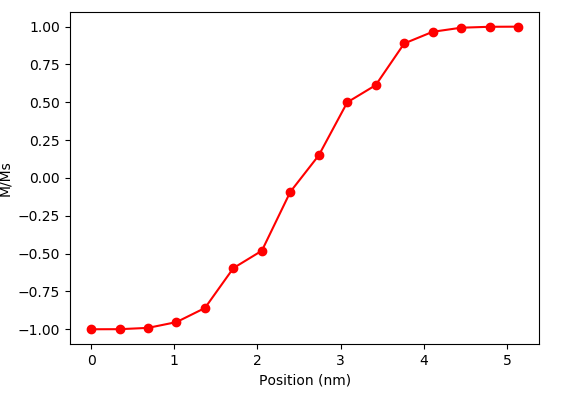 | 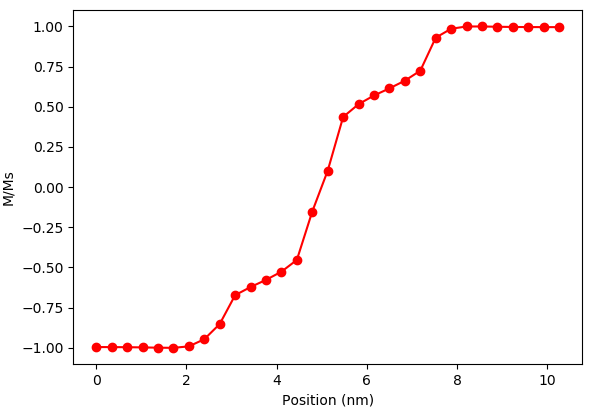 | 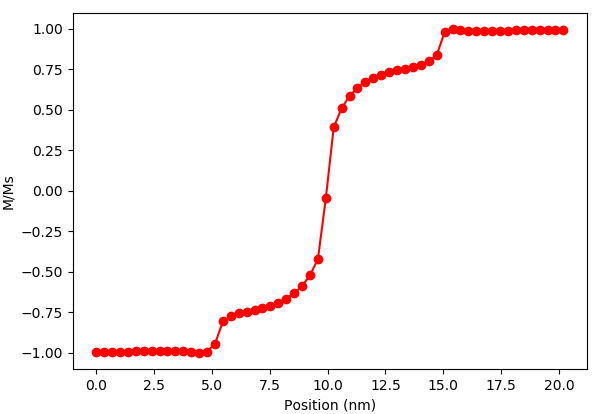 |
| 4 |  | 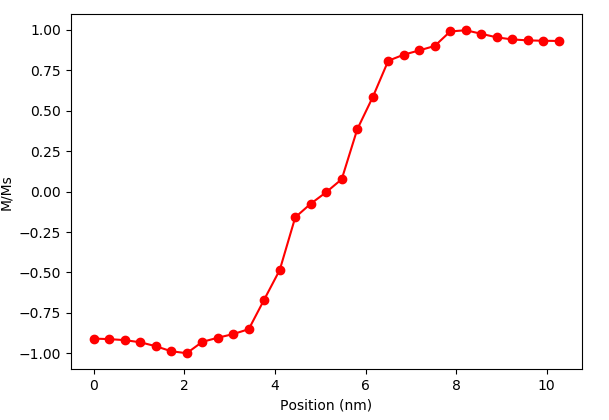 | 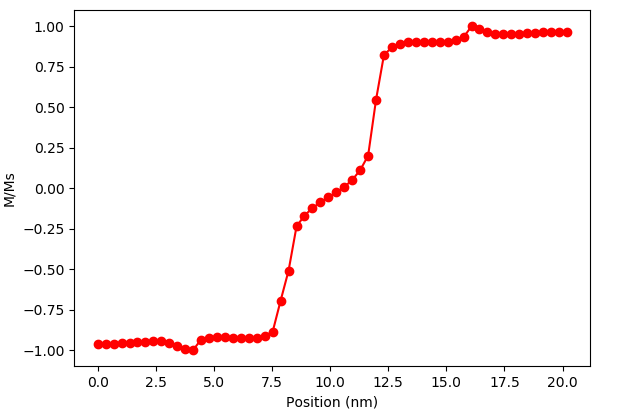 |
| 5 |  |  | 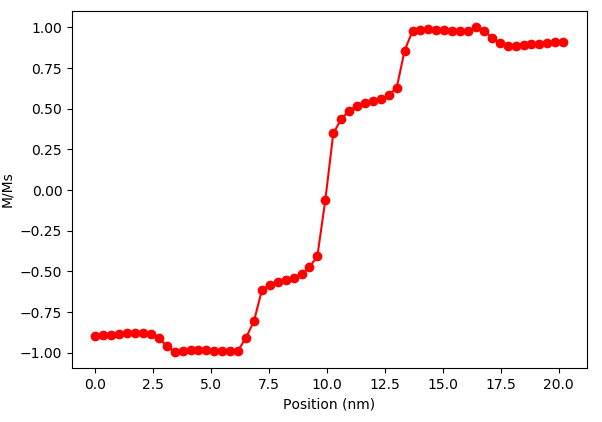 |

**Figure. S4**. $M/Ms$ profile under different grain size and FE wall number, the width of FE wall was fixed at 0.75 nm.


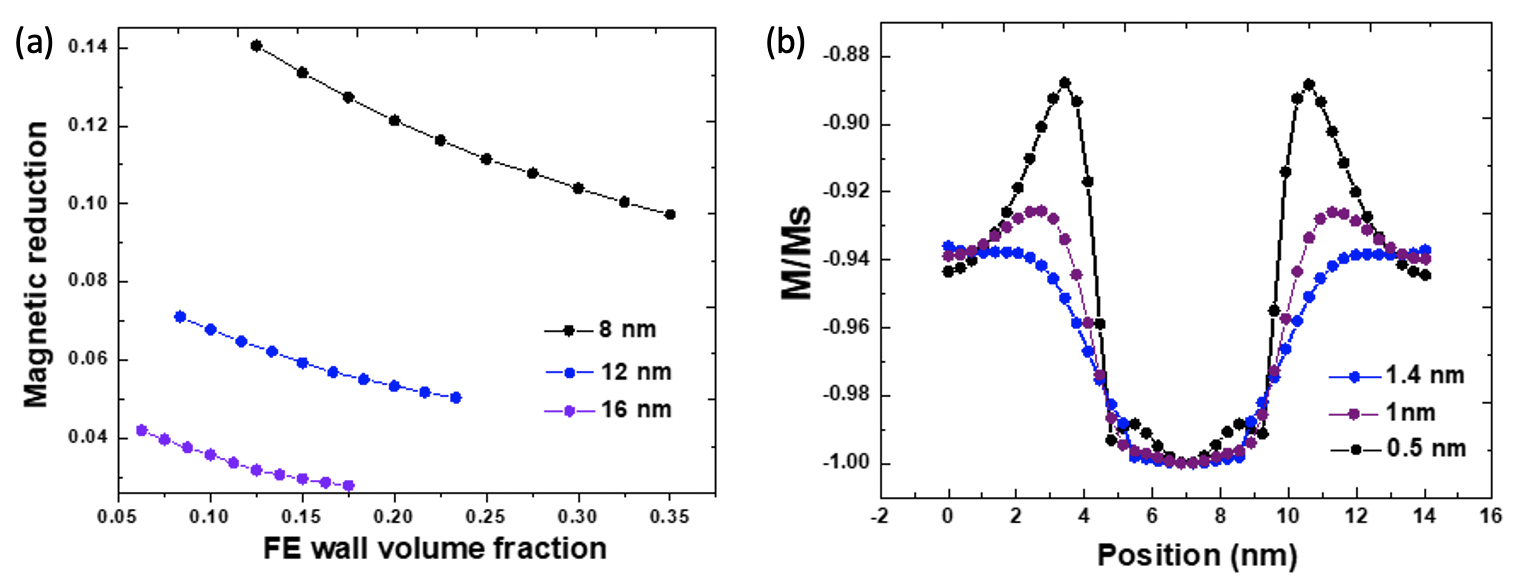


**Figure. S5** (a) The change of magnetic reduction with volume ratio of FE wall under different grain size. (b) Profile of normalized magnetization of clamped AFM wall when FE domain wall width equals to 0.5, 1 and 1.4 nm.


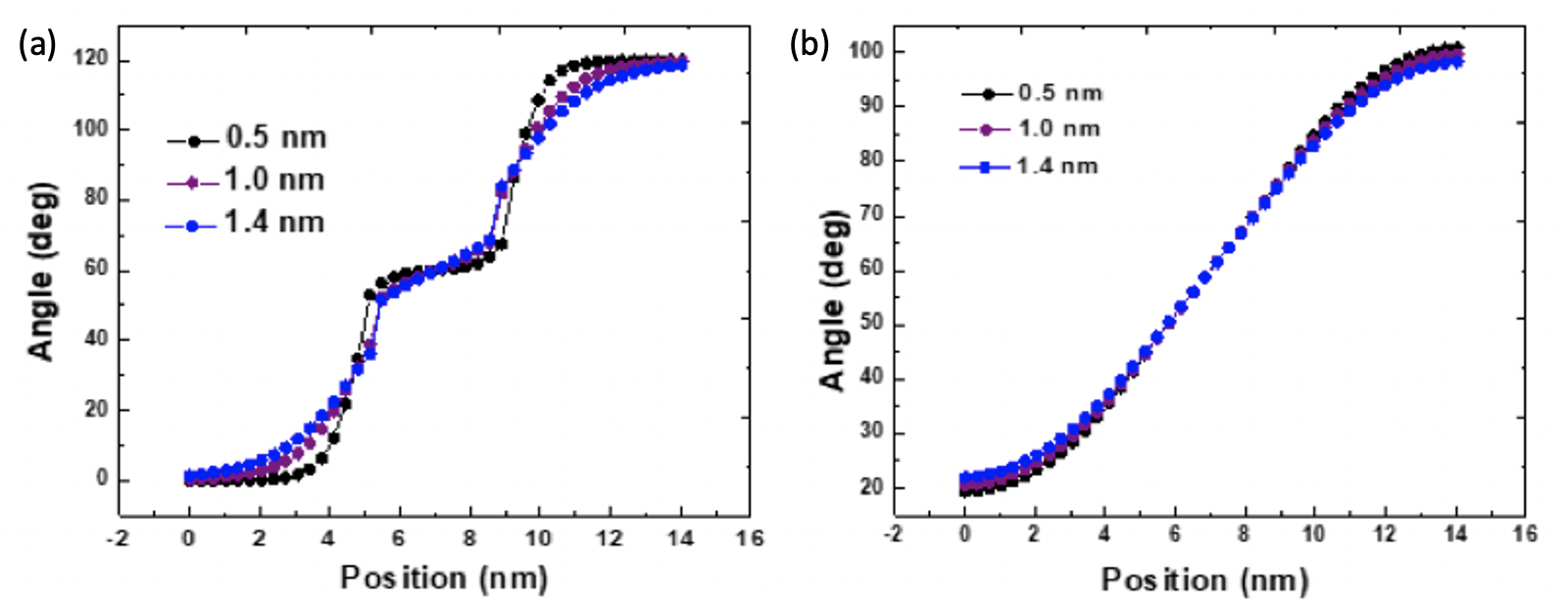


**Figure. S6** (a) Profiles of $\phi_{Q} and \left( b \right)\phi_{L}$ across clamped AFM walls when width of FE domain wall equals to 0.5, 1 and 1.4 nm.


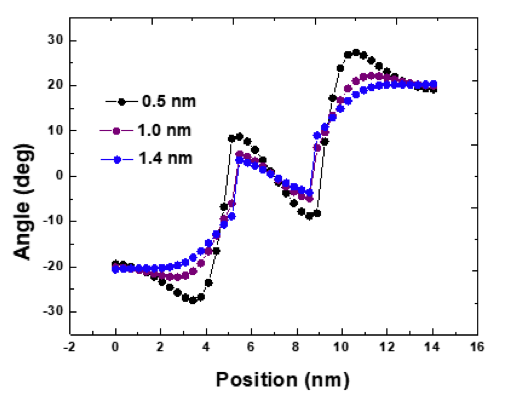


**Figure.S7** Profile of$\phi_{Q}$ $-\phi_{L}$ across clamped AFM walls when FE domain wall width equals to 0.5, 1 and 1.4 nm.

Section S3: Crystal structure characterization


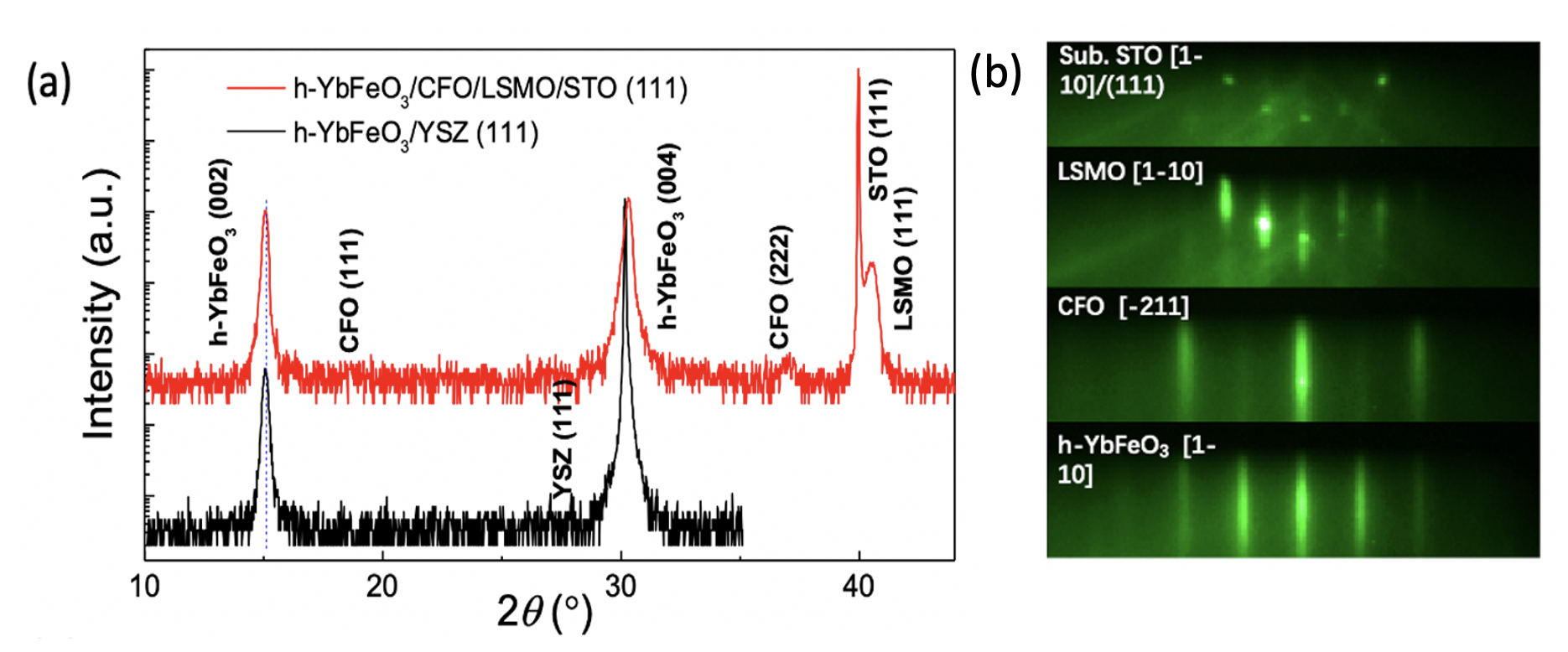


**Figure. S8.** Structural characterizations of h-YbFeO_3_ films. (a) θ-2θ XRD measurement of the films. (b) RHEED images along STO [1-10]/(111) direction.

Section S4: Quantitative analysis of HADDF-STEM images

Section S4.1: Identification of ($Q,\phi_{Q}$) basing on STEM image

To get the distribution of ($Q,\phi_{Q}$), the atom positions for individual Yb ions were first calculated through gaussian fit of HADDF-STEM images. Then based on the schematic four-lattice patterns for six FE domains in Figure. 4(a), the Q was calculated as the local maximum displacement of atoms from mean y position, and $\phi_{Q}$ was calculated as the superposition of basic patterns, using the formula:

$(y^{1},y^{2},y^{3},y^{4})=\sum_{n=0}^{5} C_{n}\cdot(y_{n}^{1} ,y_{n}^{2} , y_{n}^{3} , y_{n}^{4})$ (13)

$\phi$=$\sum_{n=0}^{5} C_{n}\cdot\phi_{n}$ with $\phi_{n}=n\cdot\frac{\pi}{3}$ (14)

in which $\left( y_{n}^{1} ,y_{n}^{2} , y_{n}^{3} , y_{n}^{4} \right)$ are normalized atom positions. Therefore, the deviation of order parameter $\phi_{Q}$ as well as FE domain walls, where $\phi_{Q}\neq\frac{n\pi}{3}$, could be represented by coefficients $\left( C_{0},C_{1},C_{2},C_{3},C_{4},C_{5} \right)$, as shown in Figure.S9.


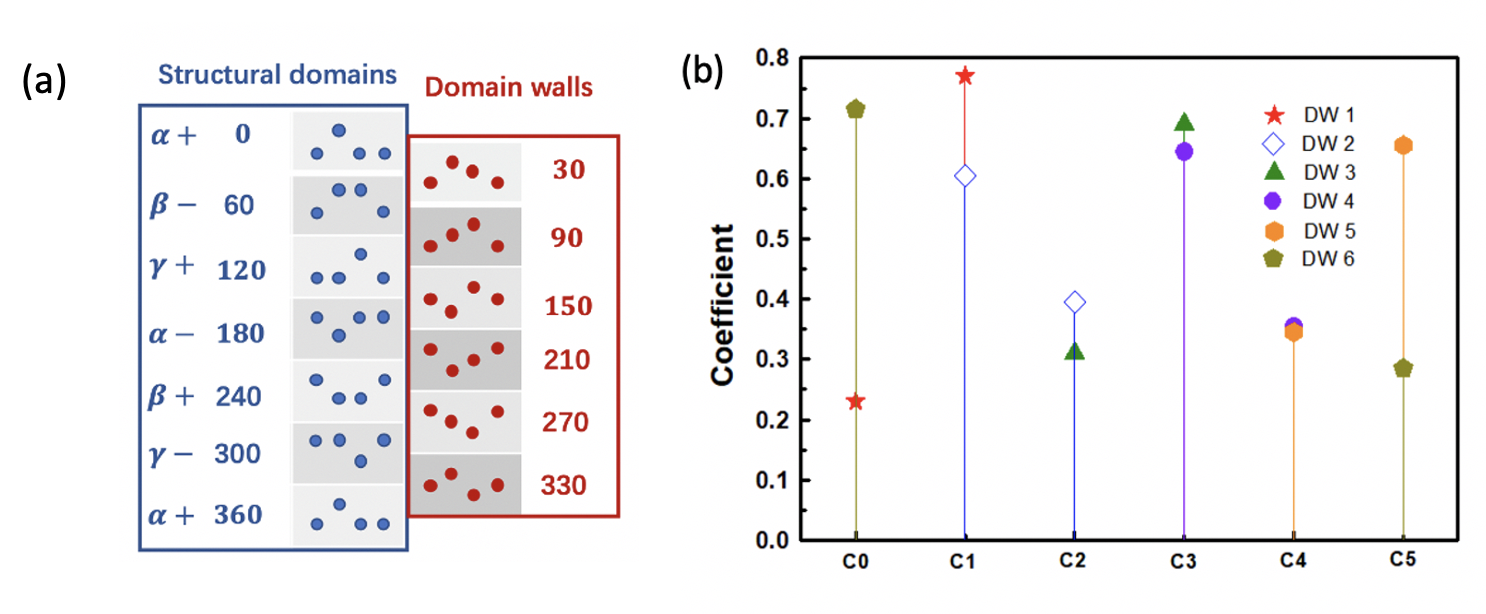


**Figure.S9** (a) Schematic diagrams of four-lattice patterns of ferroelectric domains and domains walls. (b) The coefficients ($C_{n}$) for domain walls (DW1 to DW6) in (a).

Section S4.2: Distribution of ($Q,\phi_{Q}$) in areas with single FE domain

Based on above identification mechanism, ($Q,\phi_{Q}$) distributions for single FE domain areas were investigated in Figure. S10, in which the polarization direction is up in Figure. S10 (a) and (b), and polarization is down in in Figure. S10 (c), and these areas are far away from the h-YbFeO_3_/CFO interface to preventing the influence of interfacial clamping on displacement of Yb ions. Even if without the FE domain walls, the distributions of $\phi_{Q}$in single FE domain areas still indicate high volume fraction of intermediate phase with $\phi_{Q}\neq\frac{n\pi}{3}$ , corroborating the assumption about the effect of geometrical confinement from grain size on improper ferroelectricity in h-RFeO_3_.


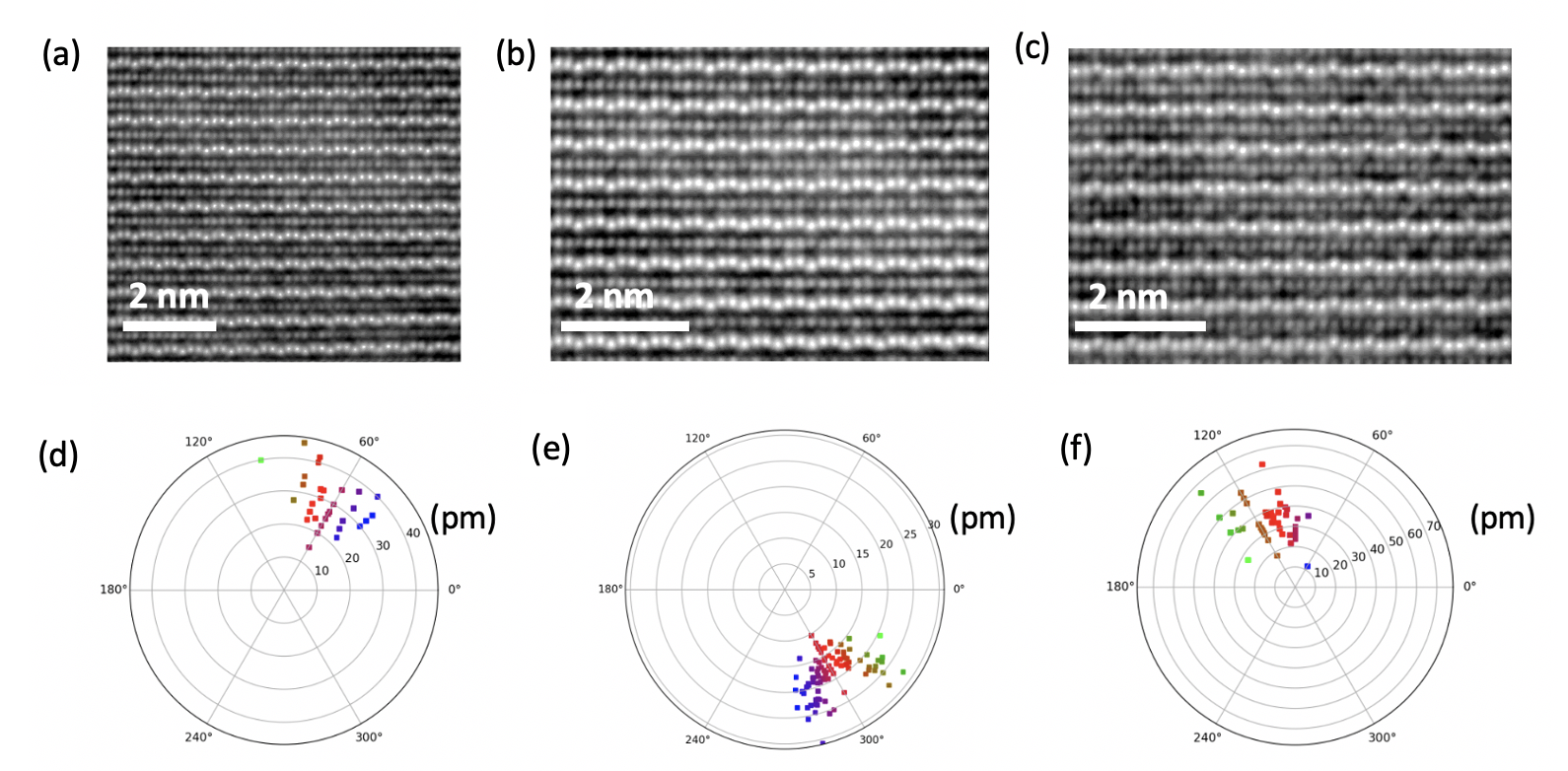


**Figure. S10** HADDF image of single FE domain area of h-YbFeO_3_ film and related (Q, $\phi_{Q}$) distribution

Section S4.3: Width of FE domain walls

The equation of FE domain walls has been derived based on Landau theory for structural energy before^31^, as:

$\phi_{Q}\left( x \right)=\phi_{0}+\frac{2}{3}\arctan\left( e^{x/\xi_{6}} \right)$, with $\xi_{6}\cong\sqrt{g/(3|c'|Q_{0}^{4})}$ (15)

in which $\xi_{6}$ serves as the characteristic length for FE domain walls and was estimated around 6 angstroms in h-ErMnO^3^. Similarly, we use this equation to fit the profile of $\phi_{Q}$ in h-YbFeO_3_ films. Figure. S11 gives an example for the domain wall fitting, in which $\xi_{6}$ equals to ﻿1.329 nm, which is larger than both widths of neutral and charge FE wall reported before.


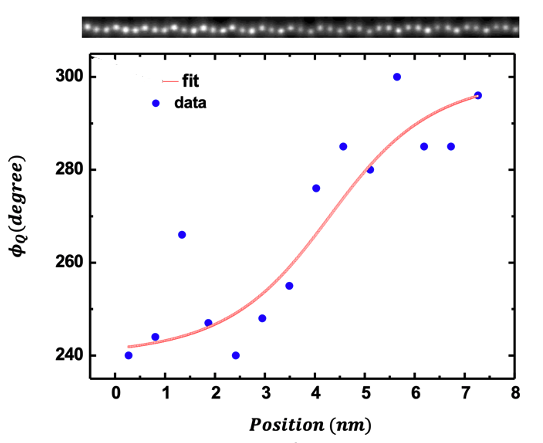


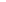

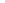


**Figure. S11** HADDF image of Yb layer in h-YbFeO_3_ film and corresponded fit for $\phi_{Q}$. The size of image is 8.067 nm*0.373 nm.

Section S5: XECD and XMCD measurements

Two mechanisms may contribute to the ME coupling in h-RFeO_3_: (1) the bulk-state ME coupling mechanism is highlighted when comparing the magnetization before and after the polarization is fully reversed since the population of domain walls is minimized. (2) the domain-wall mechanism is highlighted when comparing magnetization magnitude between saturated polarization and zero polarization states in which the domain wall population is maximized.

Therefore, two approaches have been adopted to measure ME and probe these two mechanisms: A) To probe bulk-state ME coupling mechanism, i.e., whether reversal of polarization changes magnetization, the magnetization state of the h-YbFeO_3_ is set initially using + (or -) 18 kOe magnetic field; under zero magnetic field the XAS spectra are compared before and after the polarization reversal. If the XAS contrast changes sign, the ME of electric-field controlled magnetization reversal is demonstrated. B) To probe the domain-wall ME coupling mechanism, i.e., whether the population of domain wall can change the magnitude of magnetization, the electric field is applied step by step to trace the ferroelectric hysteresis loop of h-YbFeO_3_, followed by the measurement of XMCD contrasts using +/- 18 kOe magnetic field. If the magnitude of XMCD contrast changes, the ME of electric-field controlled magnetization magnitude is demonstrated.

The approach A) can be described as the following: 1) the initial magnetization state of the h-YbFeO_3_ films was set by applying and removing + or - 18 kOe magnetic field; 2) the polarization state was set by applying and removing a positive DC voltage, followed by the measurement of x-ray absorption spectra (XAS^+^); 3) the polarization was reversed by applying a negative DC voltage, followed by the measurement of the x-ray absorption spectra (XAS^-^). The contrast is defined as XAS^+^-XAS^-^. As shown in Figure. 4c, the magnitude of XAS^+^-XAS^-^ contrast is vanishingly smaller than that of the XMCD contrast for both positive remanent (M+) and negative remanent (M-) magnetization, demonstrating that there is no obvious change of magnetization with the polarization switching.

In approach B), the polarization states were set step by step by applying and removing in-situ electric fields, followed by the XMCD measurement using the contrast of XAS under +/- 18 kOe magnetic field; this process was repeated with a sequence of applied voltages from 9 V to -9 V to 9 V to trace the polarization switching hysteresis. For every applied voltage, the XMCD contrasts were measured more than 10 times to reduce the experimental error.


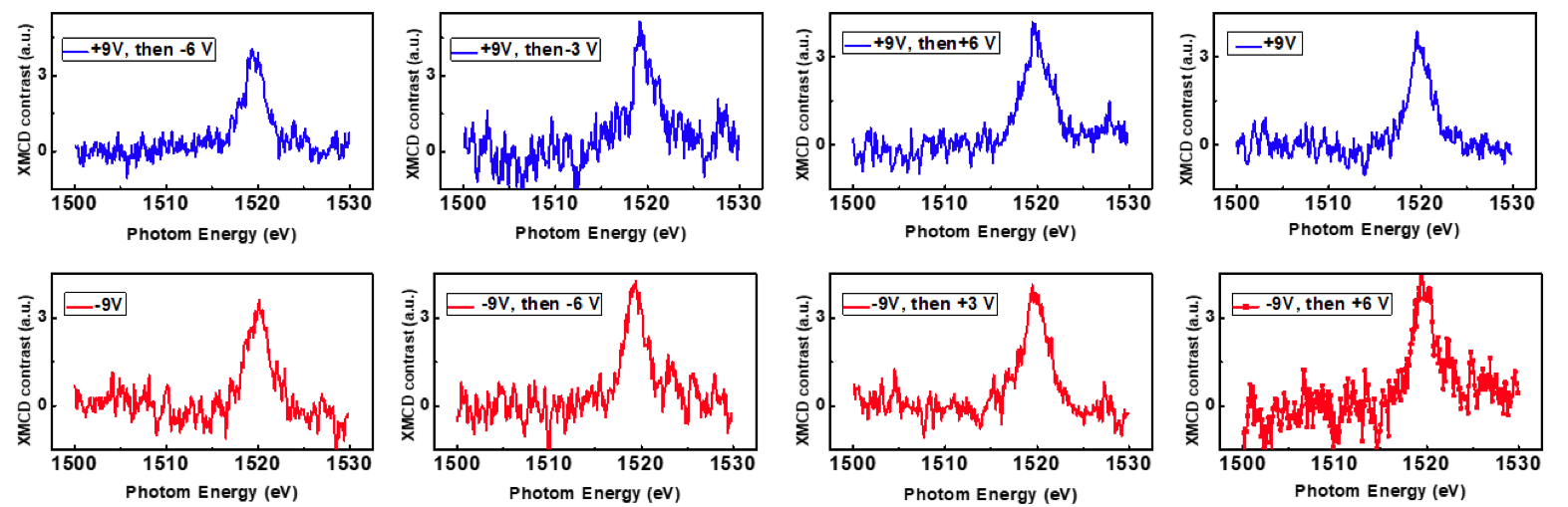


**Figure. S12** XMCD contrast of Yb at M_5_ edge with applied external voltage from 9 V to -9 V (blue) and from -9 V to 9 V (red).


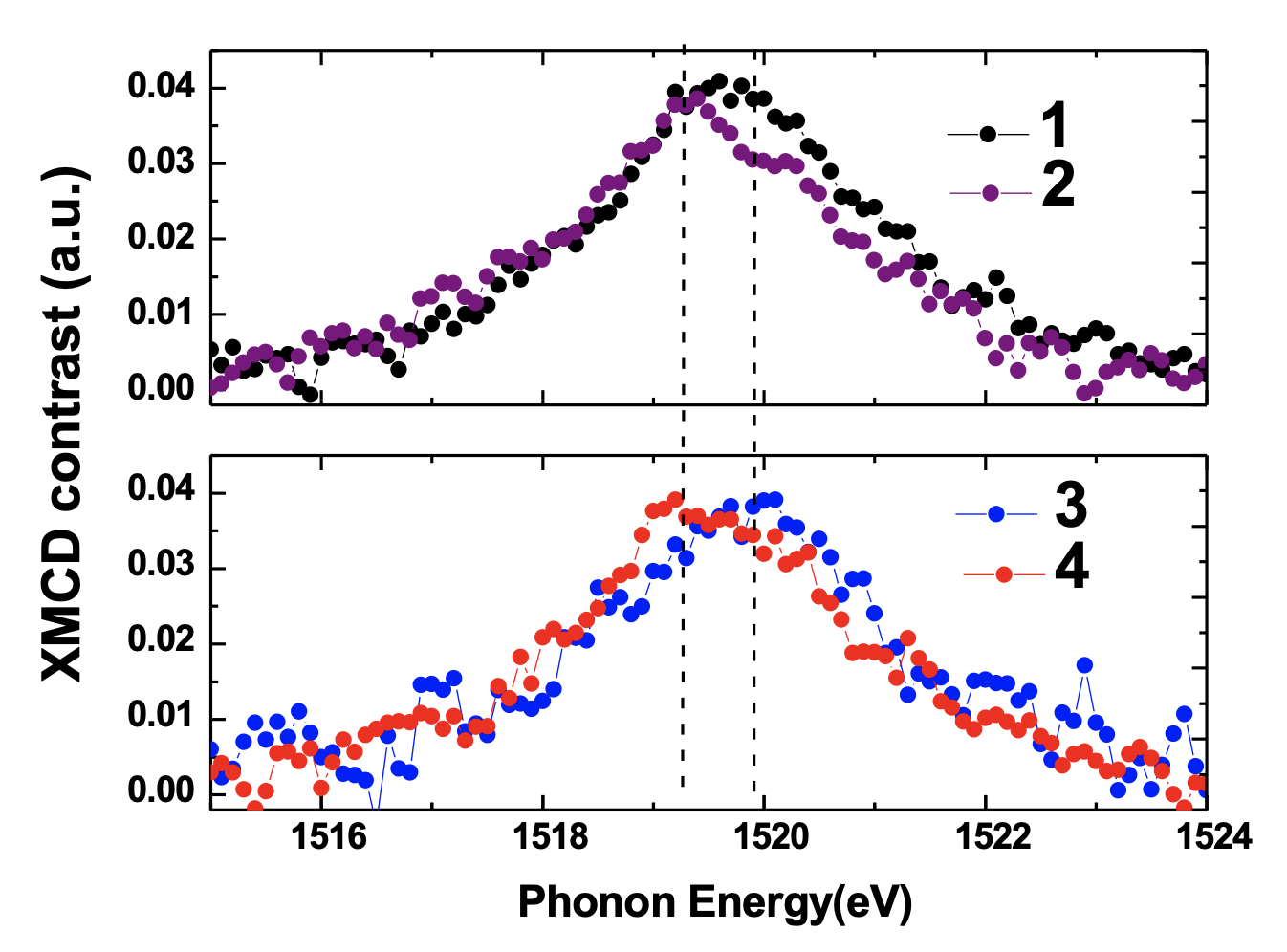


**Figure. S13** Comparison of XMCD contrast of block 1 to 4, the dash lines correspond to the estimated peak positions of saturated polarization state (block 2 and 4) and multi-domain state (block 1 and 3).

Section S6: Low temperature MFM measurements of h-YbFeO_3_/YSZ films

A commercial AFM/MFM (Atto AFM/MFM Ixs; Attocube Systems) was used to map the topography and magnetic images. During the measurement, the MFM was performed in Dual pass mode (Figure. S13) and single pass mode (Figure. S14) with PPP-MFMR tip from NANOSENSORS at different temperatures and the scan speed is 1um/s.

Section S6.1 MFM image of h-YbFeO_3_/YSZ films at 1.6K

To estimate the width of AFM walls, we use the model for 180^o^ magnetic domain wall as:

$df(x)=\mathrm{df}_{0}+\Delta f*arctan[sinh(\frac{x}{\delta})]$(16)

to fit the profile of frequency shift (df), getting from the MFM images, and resultant $\delta$ as the characteristic length from AFM walls.


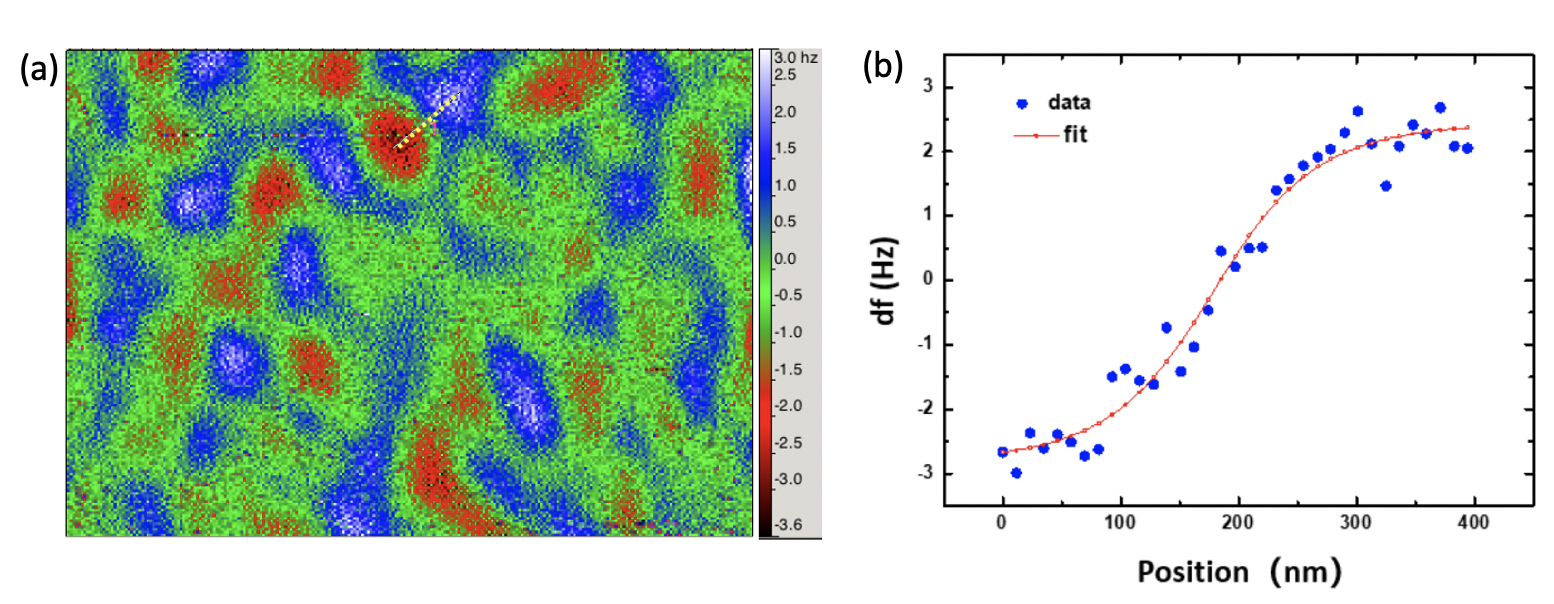


**Figure. S14** (a) MFM images of h-YbFeO_3_(001)/ YSZ(111) were measured in the phase-lock mode 30 nm above the surface. The size of the image is 3.0×2.1 μm. (b) Line profile of df along the dot line in (a) and corresponding fit.

Section S 6.2 Magnetic field driven nucleation and reversal of magnetic domains in h-YbFeO_3_/YSZ films at 12 K


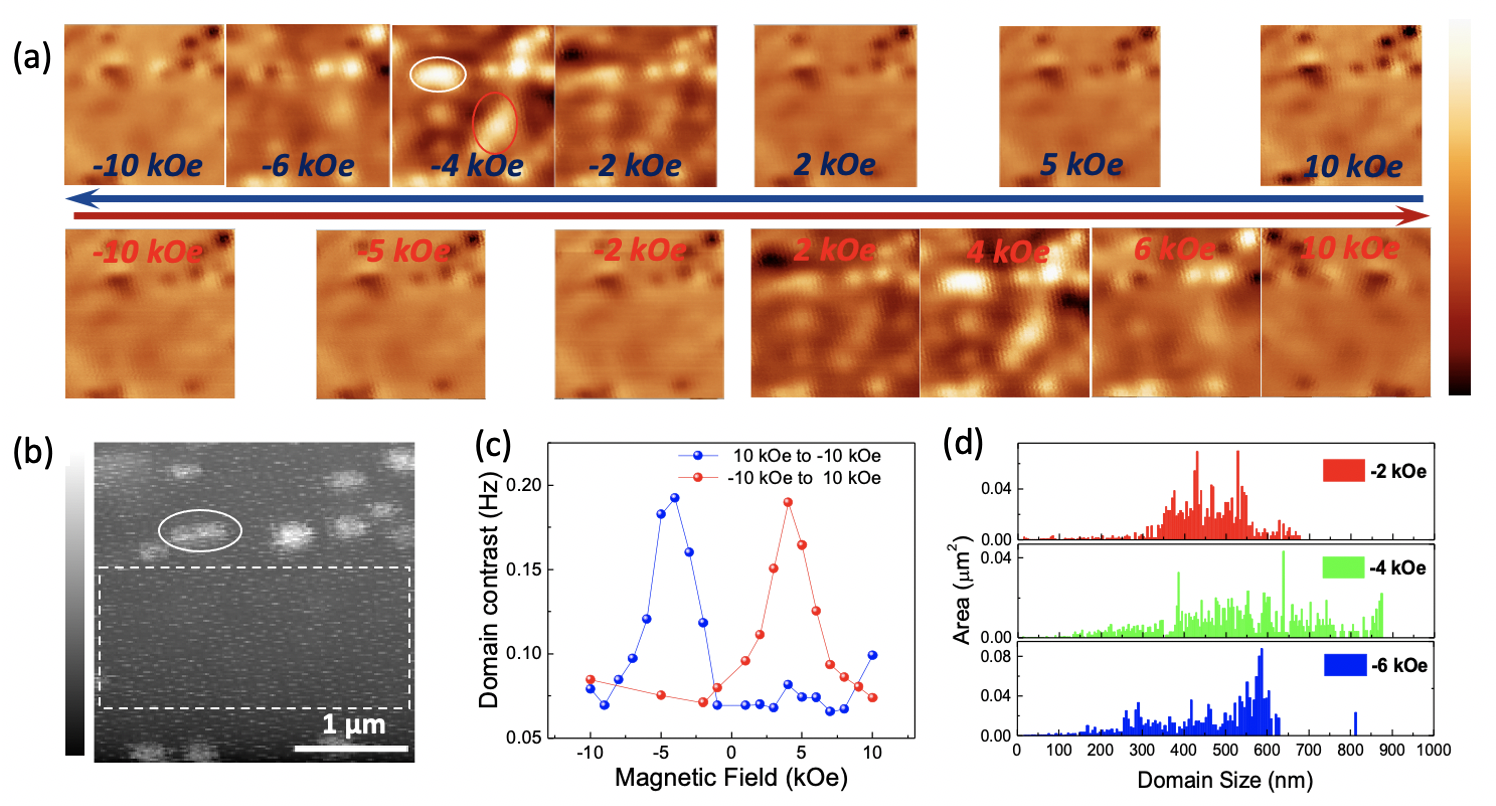


**Figure.S15** (a) MFM images of h-YbFeO_3_(001)/ YSZ(111), which were measured in the phase-lock mode 100 nm above the surface with a 3×3 μm scan area. The gray scale of the images corresponds to the frequency shift, whose full scale is 1 Hz. The arrows indicate the sequence of the applied magnetic field along the c axis of h-YbFeO_3_ films. (b) Surface topography of h-YbFeO_3_ films measured by AFM with a 75 nm full grey scale. (c) Magnetic-field dependence of the roughness of the MFM images calculated using the central part of the images in (a). (d) The distribution of magnetic domain sizes extracted from the central part of the images in (a) with reducing magnetic field. All measurements were taken at 12 K.
